# Low hunting costs in an expensive marine mammal predator

**DOI:** 10.1126/sciadv.adj7132

**Published:** 2024-05-15

**Authors:** Laia Rojano-Doñate, Jonas Teilmann, Danuta M. Wisniewska, Frants H. Jensen, Ursula Siebert, Birgitte I. McDonald, Siri L. Elmegaard, Signe Sveegaard, Rune Dietz, Mark Johnson, Peter T. Madsen

**Affiliations:** ^1^ Department of Biology, Aarhus University, Aarhus, Denmark.; ^2^ Department of Ecoscience, Aarhus University, Roskilde, Denmark.; ^3^ Department of Biology, University of Southern Denmark, Odense, Denmark.; ^4^ Biology Department, Woods Hole Oceanographic Institution, Woods Hole, MA, USA.; ^5^ Biology Department, Syracuse University, Syracuse, NY, USA.; ^6^ Institute for Terrestrial and Aquatic Wildlife Research, University of Veterinary Medicine Hannover, Hannover, Germany.; ^7^ Moss Landing Marine Laboratories, San Jose State University, San Jose, CA, USA.; ^8^ Aarhus Institute of Advanced Studies, Aarhus University, Aarhus, Denmark.

## Abstract

Many large terrestrial mammalian predators use energy-intensive, high-risk, high-gain strategies to pursue large, high-quality prey. However, similar-sized marine mammal predators with even higher field metabolic rates (FMRs) consistently target prey three to six orders of magnitude smaller than themselves. Here, we address the question of how these active and expensive marine mammal predators can gain sufficient energy from consistently targeting small prey during breath-hold dives. Using harbor porpoises as model organisms, we show that hunting small aquatic prey is energetically cheap (<20% increase in FMR) for these marine predators, but it requires them to spend a large proportion (>60%) of time foraging. We conclude that this grazing foraging strategy on small prey is viable for marine mammal predators despite their high FMR because they can hunt near continuously at low marginal expense. Consequently, cessation of foraging due to human disturbance comes at a high cost, as porpoises must maintain their high thermoregulation costs with a reduced energy intake.

## Teaser

Cheap hunting of small prey allows marine mammal predators to meet high-energy demands but requires near-continuous hunting.

## INTRODUCTION

To meet their energy requirements, predators must adopt a foraging strategy that balances the net energy gain per prey, the rate at which prey can be caught, and the time available for foraging. Within this framework, there is a range of potential foraging strategies: from low-cost hunting of abundant small prey to high-risk/high-gain pursuit of sparse, large prey. Despite this theoretical range of strategies, almost all large (>25 kg) terrestrial mammalian predators have evolved to target prey with a body size similar to their own (*1*, *2*). This pattern may be a consequence of having insufficient time to find and catch enough small prey to fulfill their large energy requirements: A lion adopting the mouse-hunting strategy of a fox will simply not be able to capture enough prey to meet its net energy needs. However, while terrestrial predators hunting large prey may gain a lot of energy per kill, the energetic pursuit and subduction lead to a transient 2- to 10-fold increase in their field metabolic rates (FMRs) (*2*, *3*), which are already elevated due to the cost of maintaining muscles, sensory systems, and cognitive capabilities to find, stalk and subdue large prey.

In contrast, many large marine mammal predators target prey three to six orders of magnitude smaller than themselves (*4*, *5*). To meet their high absolute energy requirements, these endothermic marine predators must locate, approach, and capture many hundreds, or even thousands, of small prey per day during time-constrained breath-hold dives (*6*, *7*). While baleen whales solve this challenge by bulk-feeding on dense swarms of schooling prey near the surface (*8*), toothed whales and seals consume prey one by one (*9*–*12*). This single-prey strategy is aided by exploiting foraging niches (e.g., benthic or schooling prey) and environmental factors (e.g., fronts and upwellings) that make prey more available or increase hunting efficiency. Even so, these single-prey hunters face the fundamental challenges of dedicating enough foraging time and reducing hunting costs enough to survive on tiny prey. These challenges are exacerbated for the smallest marine mammals, such as harbor porpoises (*Phocoena phocoena*) that have high mass-specific FMRs due to increased heat loss to the environment caused by a high ratio of body surface area to volume (*13**,* *14*). These metabolically expensive smaller marine mammal predators must, therefore, catch proportionally more prey per kilogram of body mass but are constrained to shorter dives than larger marine mammals, further limiting the time available for foraging at depth (*4*, *15*).

To address the question of how marine mammal predators, unlike their terrestrial counterparts, can survive on small prey, we here use multisensor biologging tags to quantify the time and energy budgets of 20 wild harbor porpoises exploiting pelagic and benthic foraging niches in shallow water. We show that despite their absolute high metabolic rates, harbor porpoises can subsist on small low-value prey by hunting at high rates for a large proportion of their time with low marginal energy costs. Nighttime foraging, which is mainly pelagic, is especially important, accounting for >70% of prey captures. Consequently, cessation of foraging due to human disturbance will come at a high cost, in particular at nighttime, as porpoises must continue to meet their high energy demands but suffer a reduction in energy intake when their near-continuous foraging is disrupted.

## RESULTS

### Diving, breathing, and foraging behavior

**Fig. 1.**
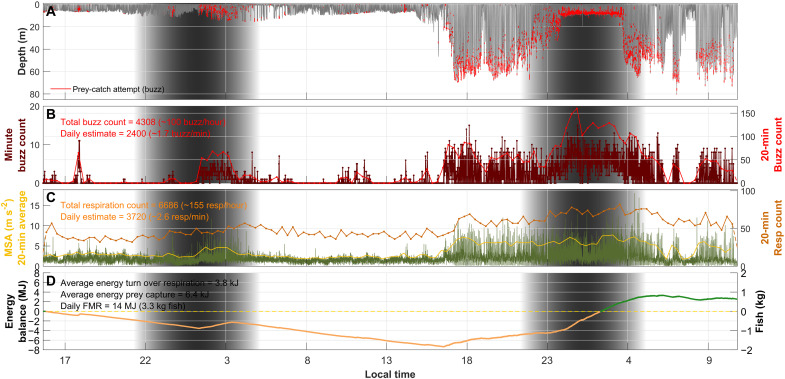
Harbor porpoise diving, feeding and breathing during a 43-hour DTAG deployment (hp18_134a, female 111 cm). (**A**) Dive profile of the harbor porpoise with prey-capture attempts (defined by echolocation buzzes) marked in red. (**B**) One-min (dark red) and 20-min (light red) buzz counts. (**C**) Respiration rates averaged over 20-min periods (orange) and 95th percentile of MSA (a proxy for activity) averaged over 5-s (green) and 20-min periods (yellow). (**D**) Energy balance calculated as the cumulative difference between the energy gained from prey captures and the metabolic energy expended (estimated via respirations). Starting from a null energy balance, 0 MJ, positive energy balance is depicted in green and negative in orange. The average energy turnover per respiration is calculated following Rojano-Doñate *et al.*, (*13*), and the average energy per prey capture is calculated using the estimated FMR of the individual [calculated following Rojano-Doñate *et al.*, (*13*)] divided by the total number of prey-capture attempts assuming a 90% assimilation efficiency. e assumed the calorific value of prey (i.e., fish) to be 4.2 kJ g^−1^ (*67*). The shaded area represents nighttime. Behavioral data for all 20 deployments are shown in fig. S9.

We used high-resolution multisensor tags [DTAGs (*16*)] on harbor porpoises to measure their diving behavior, breathing rates, a proxy for energy expenditure (*13*), and foraging behavior. Tags were attached to 20 harbor porpoises in the Kattegat and Belt seas (Danish waters) encompassing both sexes and age classes, sensu Lockyer and Kinze (*17*) (table S1). Tag recording durations ranged from 5.8 to 43.2 hours (table S1), resulting in a total of 376 hours: 191 hours of daytime and 185 hours of nighttime (from sunset to sunrise) data. Tagged porpoises spent on average 65% (SD 11%) of their time diving, mostly performing continuous short, shallow dives (table S1, Fig. 1, and fig. S9). Mean dive duration when accounting for the dependent nature of the data and autocorrelation was 61 s (ranging from 6 to 270 s), with 95% of dives shorter than 127 s (table S1). The maximum dive depth was 80 m, although 95% of dives had maximum depths of less than 25 m (table S1). Neither dive duration nor maximum depth was associated with body length or age class (see Supplementary text). The average surface time between dives was 27 s, with 95% being less than 103 s.

**Fig. 2.**
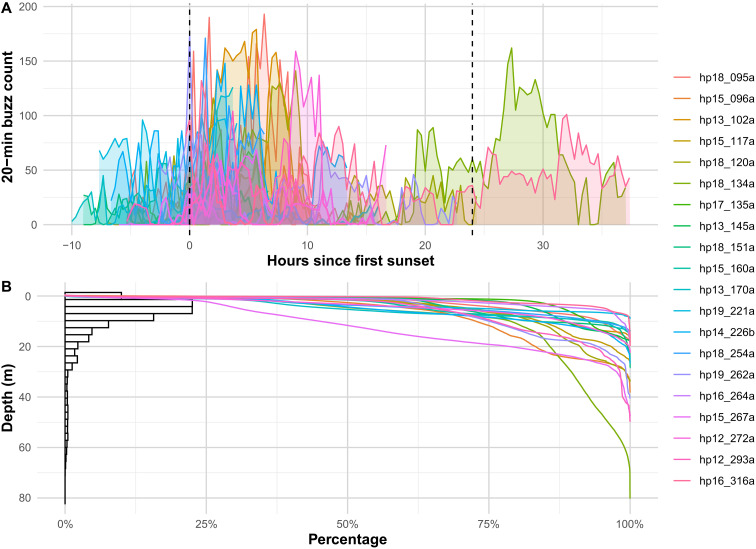
Buzz production overview. (**A**) Number of buzzes in 20-min bins as a function of time since sunset. Colored areas show the count for each tagged porpoise and the two dashed lines mark the time of first and second sunset. (**B**) Percentage of cumulative time spent above each water depth by individual porpoises (colored lines) and percentage of buzzes produced at each water depth pooling animals (bars, bin width = 3 m). The digits in the individuals’ ID indicate the year and Julian day of tag deployment and the letter indicates the order in which animals were tagged if multiple animals were tagged on the same day. Individuals are sorted by Julian day.

While the percentage of dives with feeding buzzes (i.e., prey-capture attempts) varied between tagged porpoises (range, 29 to 97%), on average, 56% of dives had at least one buzz. Dives with buzzes had a median of three buzzes, ranging from 1 to 40 (fig. S8). Buzzes were produced throughout the water column (Fig. 2B), with an overall median buzz depth of 7 m, ranging from 1 to 19 m per individual. Daily buzz rates estimated for deployments >20 hours ranged from 497 to 3784 buzzes (table S1), with median daily and hourly buzz rates being 2396 and 100, respectively. Buzz rate varied with a diel cycle; most buzzes (75%) were produced during nighttime, with porpoises producing on average 151% more buzzes per unit time [95% confidence interval (CI), 75 to 261%; *P* < 0.001] during nighttime than daytime (Fig. 2A and table S1). Hourly buzz rates varied from 0 to 371 during daytime and from 0 to 474 during nighttime (Fig. 1 and fig. S9), with an average rate of 48 and 142 buzzes per hour during daytime and nighttime, respectively (table S1).

### Behavioral states

**Fig. 3.**
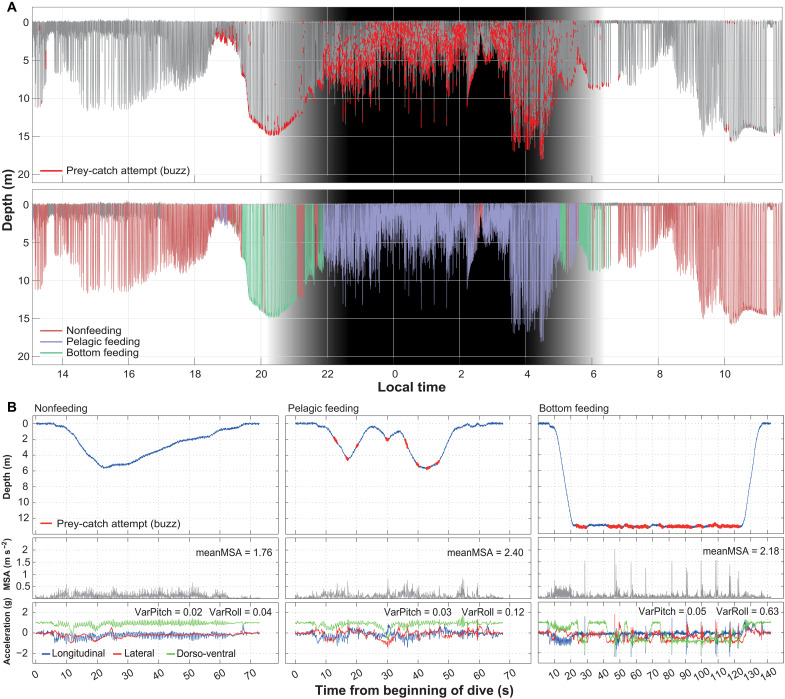
HMM results for a tagged juvenile (114 cm) male harbor porpoise (hp13_102a). (**A**) Dive profile for the complete deployment: in the top, individual buzzes are marked in red; in the lower panel, dives are color coded by their behavioral state estimated via the HMM, and apneas (see definition in Material and Methods) are in gray. The shaded area represents nighttime. (**B**) Examples of dives within each of the three behavioral states showing the dive profile with prey-catch attempts in the top, the MSA (a proxy for activity) in the middle, and triaxial acceleration along with circular variance of pitch and roll (rad^2^) in the bottom panel. HMM results for all 20 porpoises can be found in fig. S9.

Harbor porpoises hunt pelagically and benthically in the study area (*10*). As these two foraging tactics may have different payoffs, we studied how porpoises allocated time between benthic and pelagic foraging. Hidden Markov models (HMMs) on dive parameters were used to classify porpoise dives into three behavioral states: “nonfeeding” (dives with few or no buzz detections), “pelagic feeding” (with buzzes distributed throughout the dive), and “bottom feeding” (with buzzes primarily occurring near the maximum dive depth) (Fig. 3).

**Fig. 4.**
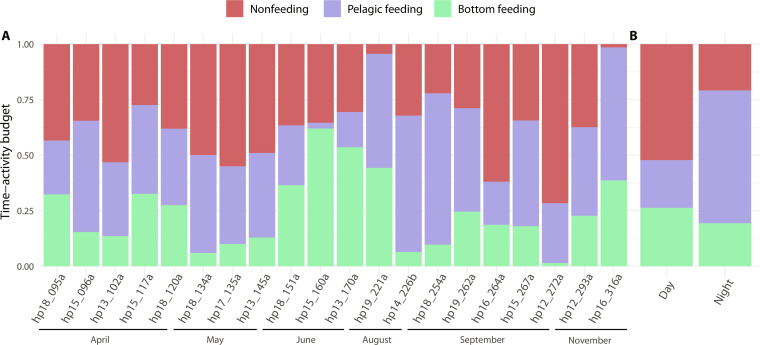
Time-activity budgets as a function of behavioral state. (**A**) Proportion of time that tagged porpoises spent in each predicted behavioral state using dive cycle (i.e., dive + subsequent surface period) as the unit. The digits in the individuals’ ID indicate the year and Julian day of tag deployment, and the letter indicates the order in which animals were tagged if there were multiple animals tagged on the same day. Individuals are sorted by Julian day to highlight potential seasonal patterns. Deployments with names in bold were longer than 20 hours. (**B**) Proportion of time spent in each behavioral state during day- and nighttime (pooling only deployments longer than 20 hours).

On the basis of the HMM states, tagged porpoises spent 62% of their diving time in foraging dives: 37% in pelagic-feeding mode and 25% engaged in bottom feeding (table S1 and Fig. 4A). Most feeding dives (65%) occurred during nighttime even though nighttime represented 49% of total deployment time. While there was no detectable difference in the probability of bottom-feeding dives between day- and nighttime (OR_ref day_ = 0.94; 95% CI, 0.67 to 1.32; *P* = 0.722), pelagic-feeding dives were more prevalent at night (OR_ref day_ = 2.38; 95% CI, 1.72 to 3.28; *P* < 0.001), and nonfeeding dives were more common during the day (OR_ref day_ = 0.47; 95% CI, 0.33 to 0.66; *P* < 0.001; Fig. 4B).

Dive duration and maximum dive depth did not change with diel cycle but were dependent on behavioral states (Table 1). Bottom-feeding dives were the longest (77.5 and 74.2 s, day- and nighttime, respectively; Table 1) and deepest dives (11.6 and 11.1 m, day- and nighttime, respectively; Table 1). While no difference in dive duration was detected between nonfeeding and pelagic-feeding dives (*P* = 0.931), pelagic-feeding dives were on average 87% deeper than nonfeeding dives (95% CI, 55 to 126%; *P* < 0.001; Table 1).

**Table 1.** Dive parameter overview as a function of behavioral state of wild harbor porpoises in the Kattegat and Belt seas. Estimates are the mean (95% CI in brackets) as calculated by the *ggemmeans* function within the *ggeffects* R package (*68*) using the generalized linear mixed models described in the methods section and assuming an average 131-cm porpoise. Respiration rate is calculated as total number of respirations in a dive cycle divided by the cycle’s duration, buzz rate is calculated as the total number of buzzes per dive cycle divided by dive duration, and meanMSA is calculated by dividing total MSA by dive duration.

	Nonfeeding		Pelagic feeding		Bottom feeding	
	Day	Night	Day	Night	Day	Night
**Dive duration (s)**	50.7 [44.2–58.1]	44.8 [39.7–50.7]	44.4 [36.1–54.8]	50.4 [43.2–58.8]	77.5 [68.9–87.2]	74.2 [64.4–85.6]
**Maximum depth (m)**	4.4 [3.4–5.8]	4.2 [3.1–5.6]	8.2 [6.5–10.4]	8.0 [6.3–10.1]	11.6 [8.9–15.1]	11.1 [8.4–14.5]
**Dive cycle duration (s)**	75.8 [66.5–86.5]	66.7 [58.7–75.7]	80.1 [67.9–94.4]	76.2 [66.8–86.9]	111.9 [100.7–124.3]	105 [93–118.5]
**Buzz rate (min^−1^)**	0.1 [0.0–0.1]	0.1 [0.1–0.3]	2.6 [2.1–3.2]	3.6 [3.1–4.3]	1.6 [1.4–1.9]	2.4 [1.9–3.0]
**Respiration rate (min^−1^)**	2.4 [2.2–2.8]	2.7 [2.4–3.0]	3.0 [2.6–3.4]	3.1 [2.8–3.4]	2.7 [2.4–3.0]	2.8 [2.5–3.0]
**meanMSA (m s** ^**−2**^ **)**	1.8 [1.6–2.0]	1.9 [1.6–2.2]	2.3 [2.1–2.6]	2.3 [2.0–2.6]	1.9 [1.7–2.2]	2.1 [1.9–2.4]

Across individuals, most buzzes (66%) were produced during pelagic-feeding dives. Pelagic feeding had the highest mean buzz rate per diving minute: 2.6 (95% CI, 2.1 to 3.2) and 3.6 (95% CI, 3.1 to 4.3), during day- and nighttime, respectively (Table 1); while mean buzz rate during bottom-feeding dives was 1.6 (95% CI, 1.4 to 1.9) and 2.4 (95% CI, 1.9 to 3) (Table 1).

### Hunting costs

Foraging dives had on average 21% higher activity (as measured by average minimum specific acceleration, meanMSA) than nonfeeding dives (95% CI, 14 to 28%; *P* < 0.001; response log-transformed). Pelagic-feeding dives were the most active, with 20% (95% CI, 13 to 28%; *P* < 0.001; response log-transformed) and 32% (95% CI, 23 to 42%; *P* < 0.001; response log-transformed) higher meanMSA than bottom-feeding and nonfeeding dives, respectively.

Postdive respiration rates (proxy for energy expenditure during diving) were positively correlated with meanMSA (*P* < 0.001; fig. S5). The 21% increase in meanMSA during foraging dives resulted in a 17% (95% CI, 11 to 23%; *P* < 0.001) increase in respiration rate. More specifically, respiration rates per minute of dive cycle (i.e., dive + subsequent surface period) were 22% (95% CI, 17 to 27%; *P* < 0.001) and 9% (95% CI, 3 to 15%; *P* = 0.004) higher after pelagic- and bottom-feeding dives compared to nonfeeding dives (fig. S7).

**Fig. 5.**
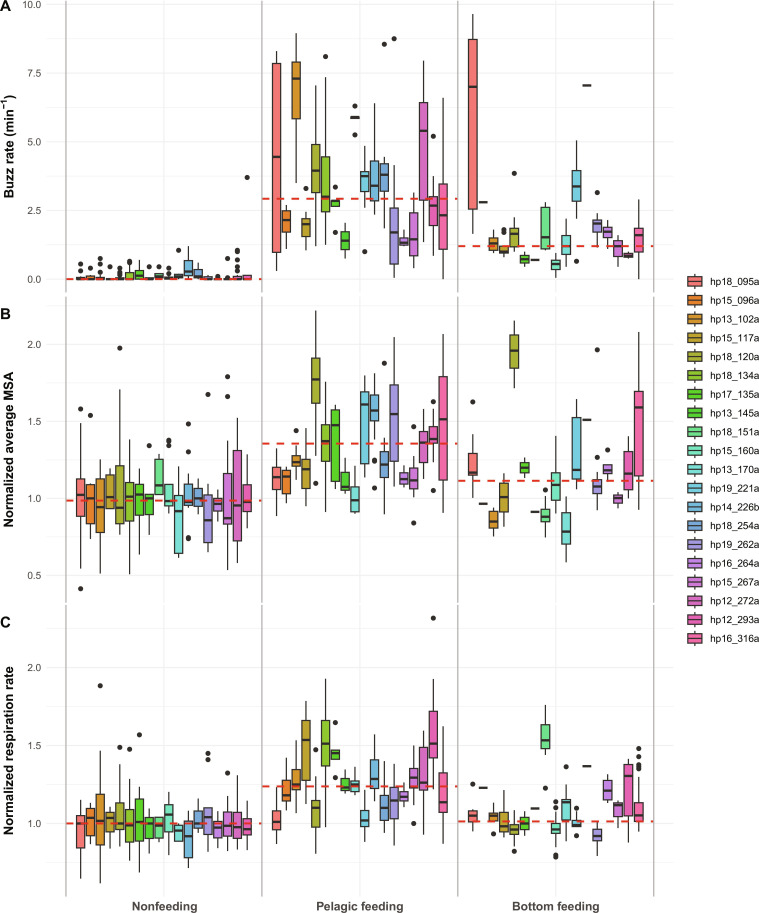
Energy proxies for each tagged harbor porpoise binned in 20-min intervals as a function of behavioral state. (**A**) Buzz rate, (**B**) normalized minimum specific acceleration (MSA), and (**C**) normalized respiration rate. Each colored box represents one of the 20 tagged harbor porpoises. Data in (B) and (C) are normalized by dividing by the median of the parameter during nonfeeding dives for each animal. The extent of each box indicates the first and third quartile and whiskers are minimum and maximum values excluding outliers (i.e., the smallest observation greater than or equal to the 25th percentile −1.5 × interquartile range or largest observation less than or equal to the 75th percentile +1.5 × interquartile range). The red dashed line represents the median of all individuals by behavioral state. Animals are sorted according to Julian day. See fig. S7 for postdive and 20-min respiration rates. The digits in the individuals’ ID indicate the year and Julian day of tag deployment and the letter indicates the order in which animals were tagged if multiple animals were tagged on the same day. Individuals are sorted by Julian day.

Smaller changes in activity and respiration rate were observed during feeding periods when analyzing 20-min intervals (Fig. 5 and figs. S5 to S7). The 20-min intervals showed 13% (95% CI, 9 to 17%; *P* < 0.001) higher total MSA in foraging intervals compared to nonfeeding intervals. This increased activity was matched by an overall 11% (95% CI, 6 to 16%; *P* < 0.001) increase in the number of respirations; specifically, a 13% (95% CI, 7 to 18%; *P* < 0.001) and a 7% (95% CI, 2 to 14%; *P* = 0.007) increase during intervals with mainly pelagic and bottom feeding, respectively (Fig. 5).

## DISCUSSION

The growing effects of climate change and anthropogenic stressors call for an understanding of the time-energy budgets of wild animals to evaluate their resilience to changing environments. However, the difficulty of simultaneously logging the behavior and metabolic rate of wild marine mammals has limited our capability to quantify the time and costs associated with different behavioral states under ecologically relevant conditions (*18*). Here, we use one of the smallest marine mammal predators, the harbor porpoise, as a model organism to address the question of how marine mammal predators, unlike their terrestrial counterparts, can subsist on prey that is three to six orders of magnitude smaller than themselves. Despite having high FMRs, we show that porpoises subsist on hunting thousands of small prey per day by using a low-cost hunting strategy. However, such a grazing strategy forces them to spend a large portion of their time foraging, making them vulnerable to repeated disturbance and changes in prey availability.

### Porpoises hunt small prey near-continuously mainly at night

The tagged harbor porpoises in the study area perform short (<2 min), shallow dives almost continuously and catch 80% of their prey at depths <15 m (Fig. 2). These short, shallow dives fit into three behavioral states: nonfeeding, pelagic feeding, and bottom feeding (Figs. 1 and 3 and fig. S9). Bottom-feeding dives are the longest and deepest, while nonfeeding dives are similar in duration to pelagic-feeding dives, but shallower and typically less active (Table 1), and are therefore likely related to traveling, nursing, or sleeping (*19*).

The tagged porpoises spend 62% of their time in foraging dives, targeting ~2000 prey per 24 hours (Table 1) mainly (66%) during pelagic feeding dives. Furthermore, porpoises have much higher foraging rates during nighttime [142 buzzes hour^−1^ compared to the 48 hour^−1^ during daytime; sensu (*10*)] and therefore engage in most foraging dives (65%) and prey-capture attempts (72%) during nighttime (Figs. 1 and 4, fig. S9, and table S1). This diel foraging pattern is consistent with passive acoustic monitoring studies that document more clicking and buzzing at night (*20**,* *21*), highlighting the importance of nighttime foraging to meet the energetic requirements of harbor porpoises in shallow waters. This elevated nighttime foraging may be related to the ease of capturing prey that are unable to see an echolocating predator in the darkness, or may result from prey moving up from the sea floor at night to feed when there is less predation pressure from visual predators such as larger fish. The latter interpretation is supported by a shift in porpoise hunting strategy toward pelagic feeding during nighttime. A light level–dependent shift in prey availability is also suggested by the tendency for increased benthic feeding during summer months (Fig. 4), when nights are 6 to 7 hours long in contrast to the 18 to 19 hours of darkness during winter months in Denmark. Such reduction in pelagic feeding may also relate to lower prey availability in open water when there is less cover of darkness in the summer months.

The high foraging rates of porpoises reported here are consistent with similarly high foraging rates recently documented in other small marine mammal predators, such as Galapagos sea lions (*Zalophus wollebaeki*) (*9*), Baikal seals (*Pusa sibirica*) (*22*), and even in larger female northern elephant seals (*Mirounga angustirostris*) (*11**,* *23*). However, the proportion of time allocated to foraging is much larger in porpoises than in these other marine predators. The 62% foraging time of porpoises also dwarfs the 36 and 40% reported for California and Alaska sea otters (*Enhydra lutris*), known for their high FMRs and foraging rates (*24**,* *25*). The very high foraging rates and large proportions of time dedicated to foraging in harbor porpoises then begs the question of whether such an intense hunting strategy incurs high costs and, therefore, contributes to their high FMRs (*13*).

### Porpoise hunting is cheap

Despite their high foraging rates, we show that during feeding compared to nonfeeding, porpoises increase energy expenditure by less than 17% when using postdive respiration rates as a proxy, and by less than 11% when computing the average respiration rate difference over 20-min of data (Fig. 5 and fig. S7). Even during pelagic feeding, the most energetic behavioral state, there is only a 22% increase in postdive respiration rates, and a 13% increase when using 20-min time bins.

To estimate the cost of hunting, we assumed that the average tidal volume and oxygen exchange fraction of respirations are the same across behavioral states and feeding rates. While some studies on captive animals report increases in tidal volume and oxygen extraction in response to longer dives or when comparing respirations after resting at the surface to after diving activity (*26**–**28*), it remains unclear to what extent porpoises, and wild marine mammals in general, systematically change these respiratory parameters during different levels of activity (e.g., nonfeeding versus feeding dives). Given that the increase in activity during foraging reported in this study is relatively low (21 and 13% increase during single dives and 20-min periods, respectively; Fig. 5B) and that the durations of foraging dives, in particular pelagic-feeding dives, are no longer than nonfeeding dives (Table 1), we argue that our estimates of the cost of hunting are unbiased by potential changes in tidal volume and oxygen extraction across behavioral states. In addition, our estimated hunting costs assume that the effect of the standard dynamic action (SDA) is evenly distributed across behavioral states. Grey seals (*Halichoerus grypus*) postpone all digestion until after periods of active foraging (*29*). In turn, porpoises in inner Danish waters do not show a strong dive response during their short breath-hold dives (*30*) and have a mean digestion time of <5 hours for a full stomach (*31*); it is therefore likely that porpoises digest during periods of active foraging (*30*). As SDA has been estimated to be 12.5% of the FMR in small marine endotherms (*32*), it follows that a porpoise will have an increase in FMR during foraging of ~24% solely due to SDA if most or all of the digestion happens during foraging bouts. If so, the small observed costs of hunting can be fully explained by the cost of digestion alone, and the actual net costs of hunting may, under this scenario, be negligible.

The small increase in FMR in hunting harbor porpoises matches the <20% increase in their postdive heart rate when foraging (*30*) and supports the conclusion that shallow-diving porpoises use low-cost foraging strategies that are akin to grazing where little effort is invested in each prey item. The increase both in activity and energy expenditure during hunting is substantially lower than reported for other marine mammal predators, including sea otters (*E. lutris*), northern fur seals (*Callorhinus ursinus*), Antarctic fur seals (*Arctocephalus gazelle*), Weddell seals (*Leptonychotes weddellii*), and large baleen whales that increase either metabolic rates or activity by at least 45% when hunting (*24**,* *33**–**36*). These numbers are in turn low compared to terrestrial predators: FMR elevations of 5.5 times the resting metabolic rate for 25-kg Wild dogs (*Lycaon pictus*) and 13 times for 170-kg African lions (*Panthera leo*) have been reported [using estimates from (*37**,* *38*)]. The low-cost foraging strategies used by harbor porpoises, and other marine mammals, resemble the strategy adopted by small terrestrial predators (<25 kg) with low absolute FMRs (*1*, *2*). However, porpoises are large predators by mammalian standards that, akin to other marine mammals, have higher FMRs than similar-sized terrestrial mammalian predators (*13*). Targeting small prey with low energetic value therefore requires them to spend a large proportion (62%) of their time foraging. This overall foraging strategy likely works for these small marine mammals because (i) in shallow aquatic environments prey are close and numerous, so little time is needed to access them; (ii) suction feeding involves a few small muscles, so it is effective and energetically cheap to subdue and ingest prey (*39*); and (iii) porpoises’ acute biosonar system provides a strong sensory advantage over their prey, allowing them to detect prey in large water volumes and forage under poor light conditions where prey are more available for capture (*40*).

### High foraging efficiency despite small prey

Animals must match their energy intakes with their cost of existence and hence use a foraging strategy that generates enough surplus energy to fuel all other life functions. Such foraging efficiency can be defined as the energy acquired while foraging divided by the marginal cost of foraging, i.e., the difference between the energy expenditure during foraging and the energy spent while not foraging (*41*). Combining the average daily FMR of 15 MJ (625 kJ hour^−1^) of a representative 40-kg porpoise [sensu (*13*)], and the average increase in postdive respiration rates of 9 and 22% for bottom and pelagic feeding in comparison to nonfeeding, we estimate the activity-specific FMR for nonfeeding, pelagic feeding, and bottom feeding to be 567, 692, and 613 kJ hour^−1^, respectively. Assuming this adult porpoise ingests 17 MJ day^−1^ (90% food assimilation efficiency) and using the estimated activity-specific FMRs, we calculate a foraging efficiency of a factor of ~9, i.e., the porpoise recoups nine times the marginal cost of foraging despite its small prey. Thus, it follows that the prey density threshold for a porpoise to engage in foraging is low, but that porpoises must capture several thousand of these per day to meet their high absolute energy needs, requiring near-continuous feeding.

Porpoises in the Kattegat and Belt seas use two main foraging strategies, with pelagic feeding being more energetically expensive than bottom feeding (Fig. 5). However, the higher foraging rates during pelagic feeding offset the increased energy expenditure resulting in a ~40% higher foraging efficiencies during pelagic feeding, assuming that prey of the same average nutritional value are caught. Benthic prey tend to be more stable and evenly dispersed than pelagic prey, while pelagic prey tend to be found in ephemeral higher-density patches (*42**,* *43*). Therefore, we posit that porpoises in the Kattegat and Belt seas opt for a more energy-demanding but energy-rewarding foraging strategy when prey are available pelagically and fall back on predictable benthic prey at other times.

### High FMR but low metabolic scopes

While there are extensive data available on the aerobic capacity of terrestrial predators, metabolic scope has only been calculated for two species of marine mammals: the harbor seal (*Phoca vitulina*) and the bottlenose dolphin (*Tursiops truncatus*) [reviewed in Williams *et al.* (*3*)]. Despite a propensity for actively chasing prey, these species have substantially lower metabolic scopes than terrestrial predators such as canids and felids (*3*) (Table 2). Small marine mammals suffer from different thermoregulatory constraints than terrestrial mammals (*14*): The higher thermal conductivity of water compared to air requires small marine mammals to elevate their resting metabolic rate, increasing heat production by making physiological (e.g., shivering and nonshivering thermogenesis) and/or behavioral adjustments (e.g., generating heat from more physical activity). An already elevated resting metabolic rate because of higher thermoregulatory costs combined with the lower cost of transport in water (*44**,* *45*) could result in a low marginal cost of hunting for many marine mammal predators despite active prey chasing. In particular, heat gained from swimming or digestion while hunting means less heat must be produced by passive means. However, even within marine mammals, porpoises exhibit a particularly low metabolic scope (Table 2).

**Table 2.** Metabolic scope of selected terrestrial and marine mammal predators estimated from their mass-specific maximum oxygen consumption (VO_2_max) and average FMR. Estimates for VO_2_ and FMR are taken from Williams *et al.* (*3*).

Species	Class	Mass-specific VO_2_max (liter O_2_/hour/kg)	Mass-specific FMR (liter O_2_/hour/kg)	Scope (factor)
Grey wolf	Canid	9.4	1.0	9.4
Coyote	Canid	11.0	0.9	12.2
Red fox	Canid	10.9	0.7	15.6
Mountain lion	Felid	2.1	0.7	3.0
African lion	Felid	3.6	0.4	9.0
Bottlenose dolphin	Marine	1.8	1.1	1.6
Harbor seal	Marine	2.0	1.1	1.8
Harbor porpoise	Marine	1.0*	0.8	1.2*

*Based on the estimated cost of hunting.

Harbor porpoises are one of the smallest marine mammals, meaning that they may have one of the highest surface-to-volume ratios and volume-specific heat loss, requiring them to use high resting metabolic rates to stay warm (*14*). The high resting metabolism and low metabolic scope, supported by the small differences between the estimated activity-specific FMRs in porpoises, further demonstrate that the FMR of harbor porpoises is high regardless of activity and behavior (fig. S6); their high FMR is likely related to being a small endotherm in cold waters that has to spend a constantly high proportion of their energy budget keeping warm.

### Perspectives and conservation implications

Environmental changes and anthropogenic disturbance have the potential to affect the time and energy budgets of animals with repercussions for their fitness and life history strategies. Information on how marine mammal predators balance their energy budgets is needed to evaluate the impact of environmental change or human disturbance (*46*).

We show that harbor porpoises in the Kattegat and Belt seas perform near-continuous short (<2 min) shallow dives to target small pelagic and benthic prey and increase feeding effort at night. With a marginal hunting cost of <20% of FMR, this foraging style is cheap. However, it requires porpoises to spend a large proportion (>60%) of their time foraging. Thus, unlike lions, porpoises can make a living by catching thousands of prey per day, each of which is about four orders of magnitude smaller than themselves.

This foraging style may reflect a dietary preference for small, easily handled prey but it could also result from a limited size range of available prey. Over the past century, commercial fisheries and eutrophication in the Kattegat and the Belt seas have markedly reduced the availability of large prey species, especially Atlantic cod (*Gadus morhua*). This may have caused a shift in the diet of harbor porpoises toward smaller fish species such as gobies (*Gobiiformes*) and sticklebacks (*Gasterosteiformes*) (*47**,* *48*). Observations of porpoises taking much larger prey in other locations demonstrate their capability to catch and ingest different prey species (*49*). However, porpoises in Danish waters may be severely restricted by the prey available, requiring continuous feeding. If so, foraging opportunities lost due to human disturbance (*50*) may be difficult to recoup by simply increasing foraging effort because of time constraints. Furthermore, given that the high FMR of harbor porpoises stems from the high cost of being a small endotherm in cold waters regardless of their activity, reductions in foraging rate due to changes in habitat and/or human disturbance are extra costly: Fasting porpoises must maintain similar FMRs to animals that are actively feeding, and they can only do this by metabolizing fat stores. Thus, frequent disturbances could result in a cumulative loss of body condition with eventual population-level consequences (*46*).

## MATERIALS AND METHODS

### Field site, animals, and tagging

The study was conducted from 2012 to 2019 in the Kattegat and the Belt seas (Denmark), which are shallow coastal seas with average depths of 23 m (maximum = 130 m) and 13 m (maximum = 81 m), respectively (*51*). We collected data from 20 harbor porpoises in good nutritional state and clinical appearance that were incidentally caught in pound nets by commercial fishermen (see (*13**,* *52*) for details). A high-resolution sound- and movement-recording tag [DTAG-3 or DTAG-4, (*16*)] was attached with suction cups approximately 5 cm behind the blowhole of porpoises. The DTAG sampled 16-bit stereo (DTAG-3) or mono (DTAG-4) audio at 500 or 576 kHz and contained a pressure sensor and a tri-axial magnetometer (sampled at 250 or 50 Hz with 16-bit resolution), as well as a tri-axial accelerometer (sampled at 625 or 250 Hz with 16-bit resolution). The tags measured 7 × 17 × 3.5 cm and weighed 221 to 321 g in air. Handling and tagging of wild porpoises were carried out under permission issued to J.T. by the Danish Forest and Nature Agency (SNS-342-00042) and the Animal Welfare Division (Ministry of Justice, 2010/561-1801) during 2012–2014; and under permissions from the Environmental Protection Agency (Ministry of Environment and Food of Denmark, NST-3446-0016) and the Animal Experiments Inspectorate (Ministry of Environment and Food of Denmark, 2015-15-0201-00549) during 2015–2019.

### Data processing

Data processing was performed with custom-written scripts in MATLAB (R2021b, MathWorks). Pressure, magnetometer, and accelerometer measurements were converted to depth (m), magnetic field (μT), and acceleration (m s^−2^), respectively, and were then decimated to a standard sampling rate of 25 Hz [www.animaltags.org; (*53*)].

Following Rojano-Doñate *et al.* (*13*), we removed the first hour of each deployment to reduce the potential effect of animal handling on the results. Respirations and buzzes [i.e., biosonar-based prey-capture attempts; (*54*)] are detectable in porpoise DTAG data as the tag is placed close to the blowhole (fig. S10). Respiration and buzz detections were automated using supervised detection algorithms. Respirations were initially detected by finding independent surface periods above a maximum depth criterion of 0.5 m and defining the respiration time as the lowest pressure value within each surfacing, keeping a minimum inter-respiration interval of 0.4 s (i.e., minimum inter-respiration interval in manually audited deployments). To detect foraging buzzes, we ran a customized click detector. Acoustic recordings were bandpass filtered with a four-pole Butterworth filter between 100 and 240 kHz, and the signal envelope was calculated by taking the absolute value of the Hilbert transform. Individual clicks were identified using a peak detector with a dynamic intensity threshold (*55*) based on in-band background noise, with a minimum threshold of −60 dB relative to tag clip level. To ignore surface reflections, peaks within 1 ms after a detection were dismissed. Potential buzzes were defined as click series with inter-click intervals shorter than 10 ms for at least five consecutive clicks that lasted at least 0.2 s and contained >100 clicks. Automatic detections of respirations and buzzes were verified using aural and visual examination of recordings. Spectrograms of 5-s segments of audio data (Hamming window, Fast Fourier Transform size 512, 75% overlap) were displayed alongside the corresponding dive profile with buzz and respiration detections overlaid (fig. S10). Validated buzzes with <1-s separation were combined into a single buzz to reduce the probability of double-counting prolonged chases.

### Dive detection and behavioral state classification

From the 20 deployments, we detected a total of 68437 interbreath intervals (IBIs; i.e., the time between consecutive respirations) that included actual dives and near-surface short submersions (hereafter, apneas) that reflect the animals’ need to take repeated breaths. To distinguish actual dives from apneas, we used a Gaussian multivariate mixture model [*Mclust* in the package *mclust* version 5.4.10 (*56*) in R version 4.1.2 (*57*)] on log-transformed IBI duration (s), maximum depth (m), and average MSA (m s^−2^; hereafter, meanMSA; a proxy for average IBI activity). MSA was calculated following Simon *et al.* (*58*) and values above the 95th percentile were trimmed to reduce the effect of transient spikes. The group with IBIs of the longest duration, the deepest depth and highest activity, a total of 14290 IBIs, was classified as actual dives (hereafter, dives) (see Supplementary text and figs. S1 and S2 for details).

Porpoises in the Kattegat and Belt seas display different foraging strategies (*10*). Using parameters related to the movement and foraging behavior of the tagged porpoises for each dive, we used HMMs to classify dives into potentially different behavioral states. Following Isojunno *et al.* (*59*), the models included discrete-valued random effects to account for differences between tag records, allowing the transition probabilities of each individual to derive from 1 to 4 common behavioral contexts with a unique transition probability matrix (TPM) (*59**–**61*). Each model was fitted 50 times with different initial values to increase the chances of finding a global minimum (*59*). Model selection was based on information criteria, choosing the model with the best goodness of fit estimates (i.e., Akaike information criterion) as the most parsimonious (*59*).

The best-fit model used eight dive metrics to estimate three behavioral states (fig. S3). The final metrics (and their parametric distributions) were: (i) log-transformed meanMSA (Gaussian), (ii) median buzz depth relative to maximum dive depth (beta), (iii and iv) circular variance of pitch (beta) and roll (beta), (v and vi) proportion of time at the bottom (beta) and surface (beta), and (vii) circular variance of pitch at the bottom (beta) and (viii) present/absence of a buzz (Bernoulli). Pitch and roll (rad) were estimated following (*53*). Circular variance (rad^2^) was calculated using the function *circ_var* from the Circular Statistics Toolbox (*62*). Times at the bottom and surface were calculated as the proportion of the dive the animal spent within one body length of the maximum depth and two body lengths from the surface, respectively. The best results were obtained when fitting either three or four states. Comparing dive-state classification to buzz distribution over individual dive profiles showed that both models identified two foraging modes, and either one or two nonfeeding states, so we selected the simpler and more parsimonious three-state model as our final model. The state-dependent distributions and dive classification of the best-fit model were similar to those of the model with three behavioral states and different number of behavioral contexts, confirming that the main differences between these models were in the transition probabilities, not the properties of the states themselves (*60*). Consequently, we selected the model with only one behavioral context as the most parsimonious alternative [i.e., explaining variation in state transition probabilities was not the primary objective of this study (*63*)]. The probability of transitioning from one state to another was summarized by the TPM (table S2).

### Energetic cost of behavioral states

Proxies for energy expenditure were quantified over each dive cycle, defined as the interval between the start of a dive and the surface time until the start of a successive dive. Average dive activity (i.e., meanMSA) and respiration rate in each dive cycle (i.e., number of respirations between successive dives divided by the dive-cycle duration) were used to calculate the relative costs of different behavioral states as a function of the nonforaging behavioral state. In addition, energy metrics were averaged over 20-min intervals (i.e., equivalent to about 10 dive cycles) to control for potential cost spillover between subsequent dives (*33*). The behavioral state of each 20-min interval was taken as the behavior that occupied most of the time in the interval. Intervals were only analyzed if the porpoise engaged in the same behavior for >75% of the interval to reduce the probability of quantifying the cost of mixed behaviors. This conservative approach led to the exclusion of 29% of intervals (792 of 1122 intervals were retained); however, an analysis using all intervals as a robustness check rendered similar results.

### Statistical analysis

Given the strong correlation between the dive variables, we used causal diagrams (fig. S4) (*64*) to assess which variables were potential confounders and which ones to include in each regression model. We used generalized linear mixed models [*lme* in the *nlme* package version 3.1-153 (*65*)] and *glmer* in the *lme4* package version 1.1-28 (*66*) in R to account for the dependent nature of dives coming from the same animal. All models included animal ID as a random intercept and the estimated behavioral state and its interaction with diel cycle as a random slope within animal ID, as well as an autoregressive covariance structure of order 1 to account for the temporal autocorrelation of the data. A Gaussian family function was used for most response variables and a log transformation was applied when the assumptions of normality or homoscedasticity of residuals were not met. A Poisson (link = log) family function was fitted when the response variable represented counts, and included an offset accounting for the log-transformed duration of the dive or dive cycle when estimating buzz or respiration rates, respectively. A binomial (link = logit) family function was fitted for dichotomous outcomes and odds ratio (OR) was used as a measure of association. Model results are reported by an estimate (α, in the unit of each parameter or OR), its 95% CI and a *P* value.

## Supplementary Materials

### This PDF file includes:

Supplementary Text Figs. S1 to S10 Tables S1 and S2 References
