# Supplementary material for: Low hunting costs in an expensive marine mammal predator
Source: Sci Adv. 2024 May 15;10(20):eadj7132. doi: 10.1126/sciadv.adj7132 (PMC11318689; doi:10.1126/sciadv.adj7132)
Supplement: Supplementary file 1 — Supplementary Text Figs. S1 to S10 Tables S1 and S2 References [file sciadv.adj7132_sm.pdf]

Science Advances
AAAS

Supplementary Materials for

Low hunting costs in an expensive marine mammal predator

Laia Rojano-Doñate *et al.*

Corresponding author: Laia Rojano-Doñate, laia.rojano.donate@gmail.com

Sci. Adv. **10**, eadj7132 (2024)
DOI: 10.1126/sciadv.adj7132

This PDF file includes:

Supplementary Text
Figs. S1 to S10
Table S1 and S2
References

Multivariate mixture model to distinguish actual dives from regular apneas.

Dive thresholds have been typically defined *a priori* depending upon dive depth or duration. However, a preliminary exploration of the data demonstrated that this traditional definition was poorly suited to the tagged porpoises with extremely short and shallow dives, and foraging at all depths. We used a gaussian multivariate mixture model (*Mclust* in the R package *mclust* (version 5.4.10 (*58*)) on the log-transforms of inter-breath interval (IBI) duration, depth and average activity (i.e., meanMSA) to distinguish *actual dives* from *apneas*. We tested multiple scenarios for the number of clustering groups, where the most parsimonious model (highest log-likelihood) classified IBIs into 3 groups (Fig. S1). The group with IBIs of the longest duration, the deepest depth and highest activity, encompassing 14290 IBIs, was classified as actual dives, while the remaining two groups, likely comprising regular apneas (n = 47232) and logging periods (n = 6915) were classified as apneas.

Exploration of the apneas, both regular apneas and logging periods, demonstrated that the median duration and maximum depth of the apneas were 9 s and <1 m, with 95% of them being less than 20 s and shallower than 2 m (Fig. S2). Apneas had low circular variation of the roll (median = 0.01, Inter-Quartile Range = 0.02) compared to the actual dives (median = 0.12, Inter-Quartile Range = 0.22) (Fig. S2), and less than 3% of them contained buzzes.

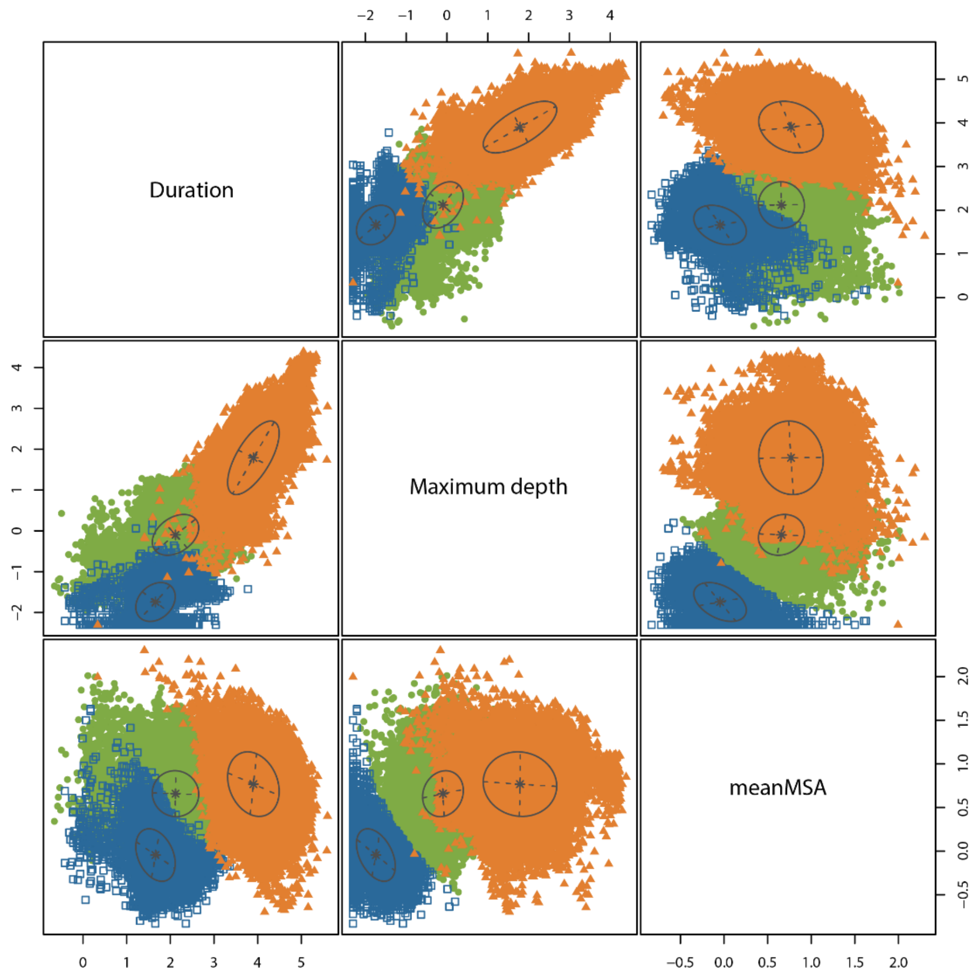

Fig. S1. Scatter plots of the natural log-transformed inter-breath intervals (IBI) duration, maximum depth and average activity (meanMSA) for the 20 free-ranging harbor porpoises. The color and shape depict the results of the best multivariate mixture model. Centroids of each group are [3.90 1.79 0.77] (orange), [2.11 -0.10 0.66] (green) and [1.66 -1.74 -0.04] (blue). The group with IBIs of the longest duration, the deepest depth and highest activity (orange) was classified as *actual dives*, while the remaining two groups (green and blue) were classified as *regular apneas* and *logging periods*, respectively.

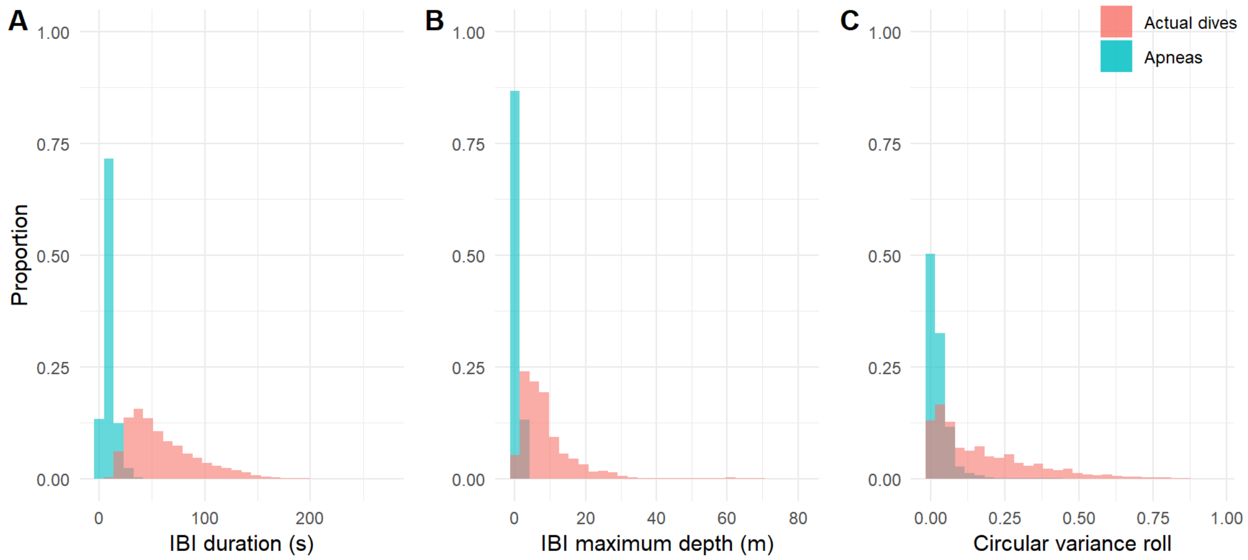

Fig. S2. Proportion of IBI as a function of IBI category (apneas vs. actual dives). A) IBI duration, B) maximum depth, and C) circular variance of the roll of free-ranging harbor porpoises as a function of IBI category: *apneas* (n = 54147) and *actual dives* (n = 14290).

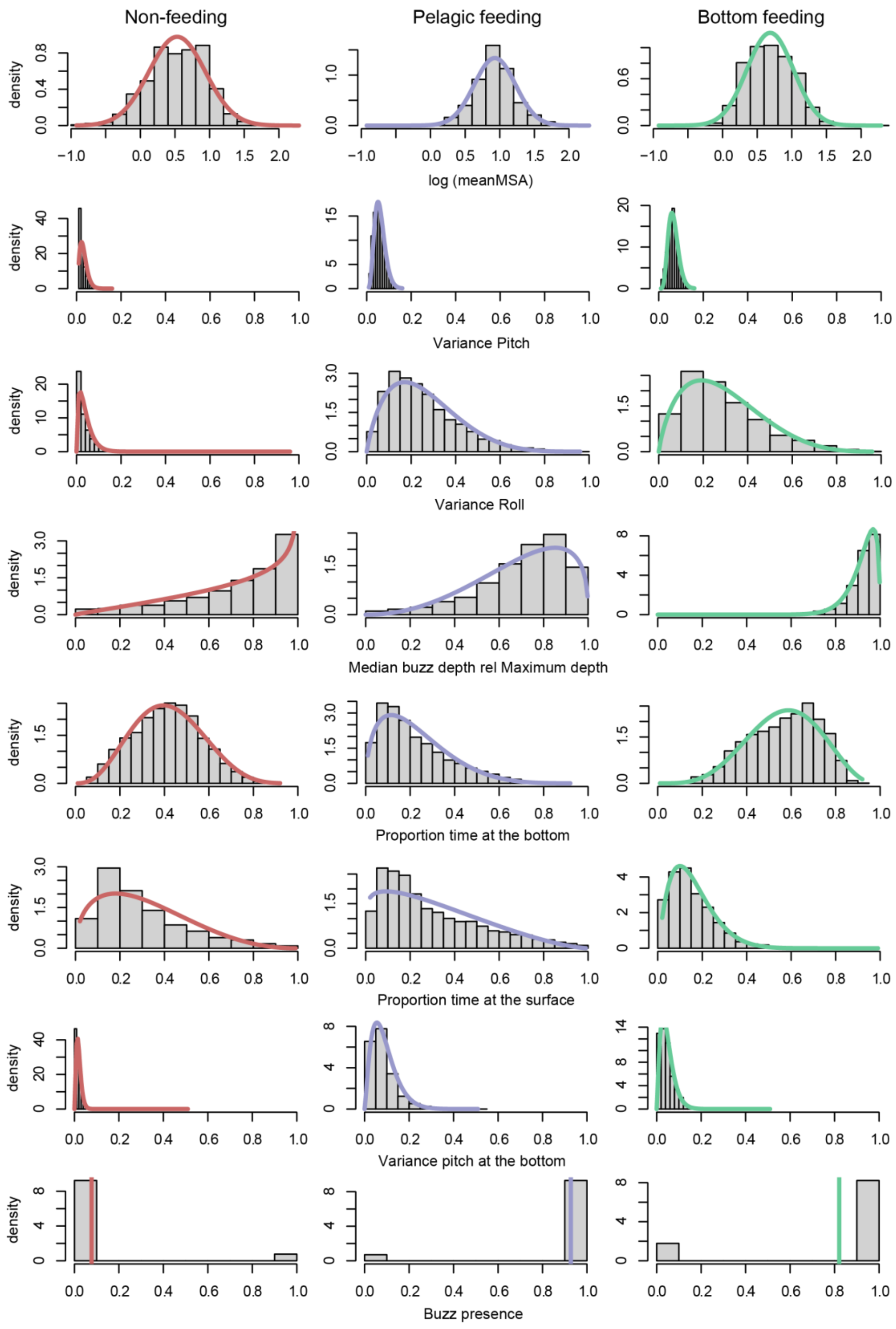

Fig. S3. State-dependent distributions estimated from the best Hidden Markov Models (HMM) for categorizing dive types of harbor porpoises from Danish waters.

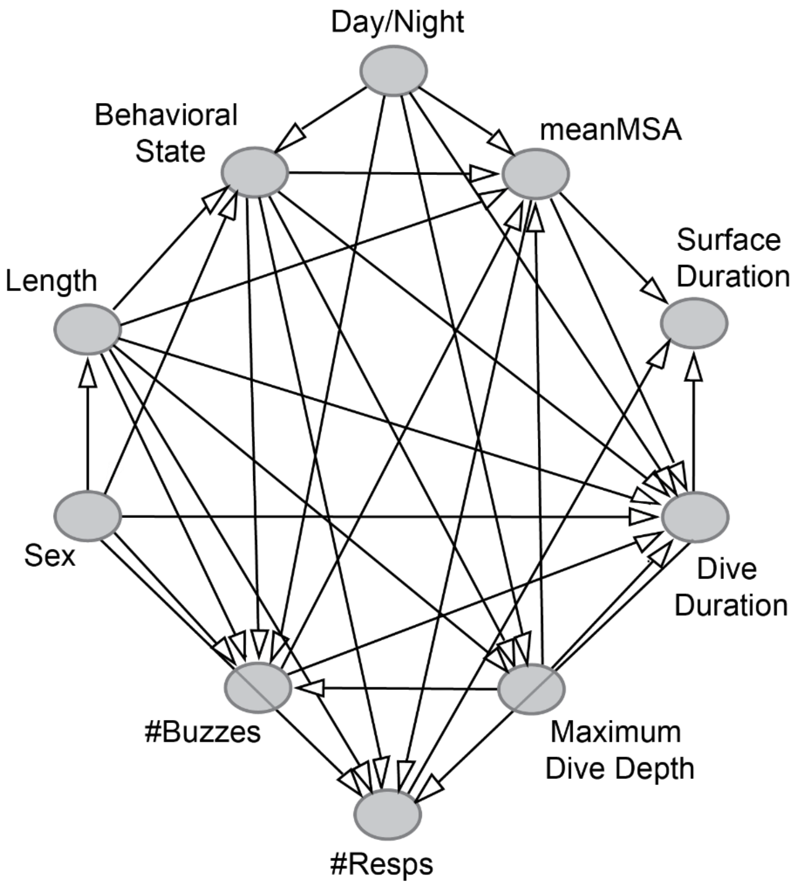

Fig. S4. Causal diagram or Directed acyclic graph (DAG). Causal relationship of the variables used in the study (built through dagitty.net using Textor et al. (*69*). In a DAG, variables are depicted as nodes and connected by arrows referring to the direction of the causal relationship. DAGs contain both causal and non-causal pathways: a causal pathway has an arrow pointing away from the main explanatory variable going towards the outcome; a non-causal pathways connect the main explanatory variable and outcome via a pathway with at least one arrow directed against the flow of time. For more details see Hernán and Robins (*66*).

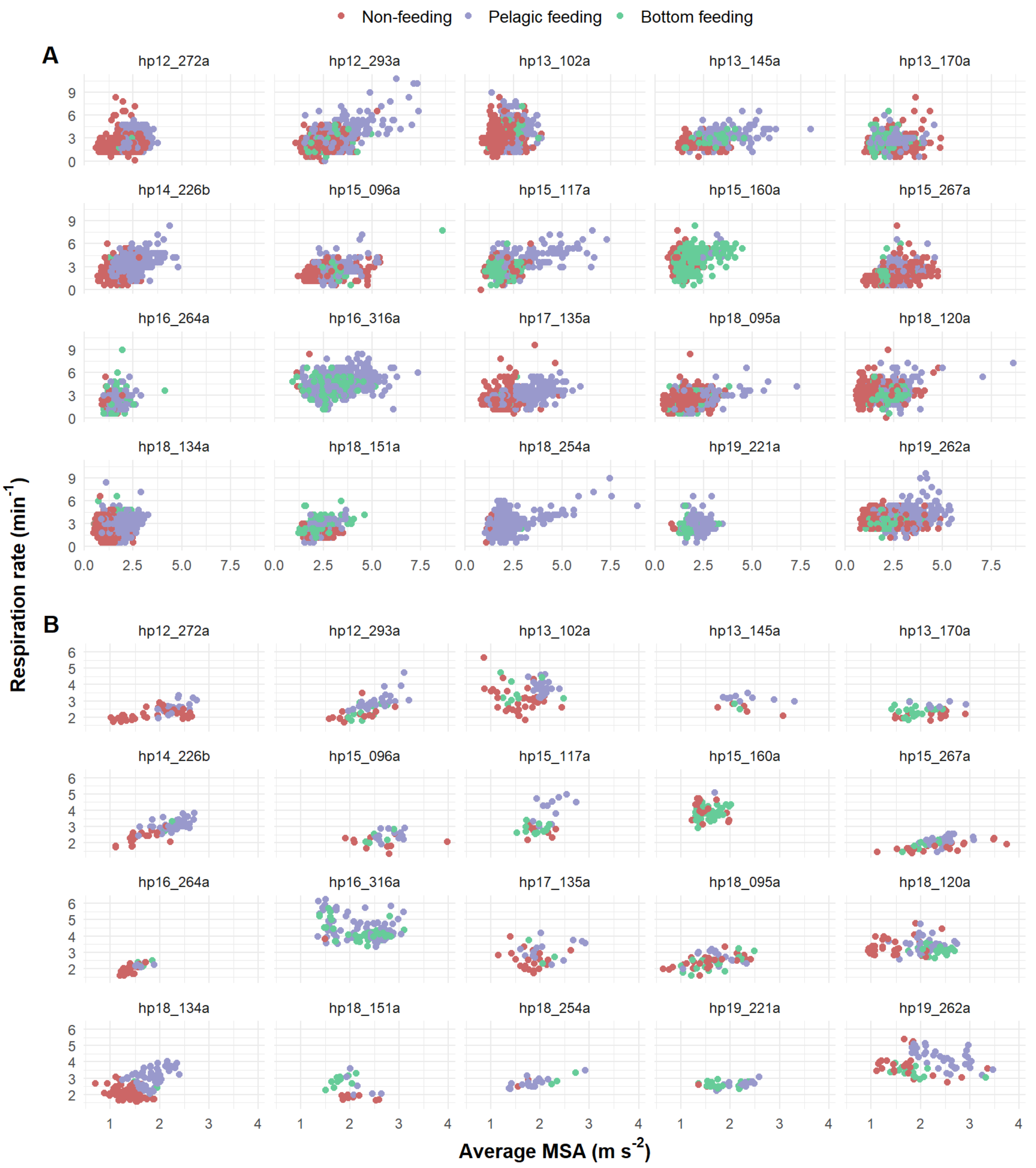

Fig. S5. Respiration rate as a function of average activity of the lower 95th percentile of MSA (meanMSA) of the 20 tagged harbor porpoises from the Kattegat and Belt Seas summarized A) post-dive and B) in 20-minute intervals. Dots are color-coded by behavioral state. Note that we chose to use 20-minute bins as they correspond to 10 times the 95th percentile of the dive cycle duration of the tagged porpoises.

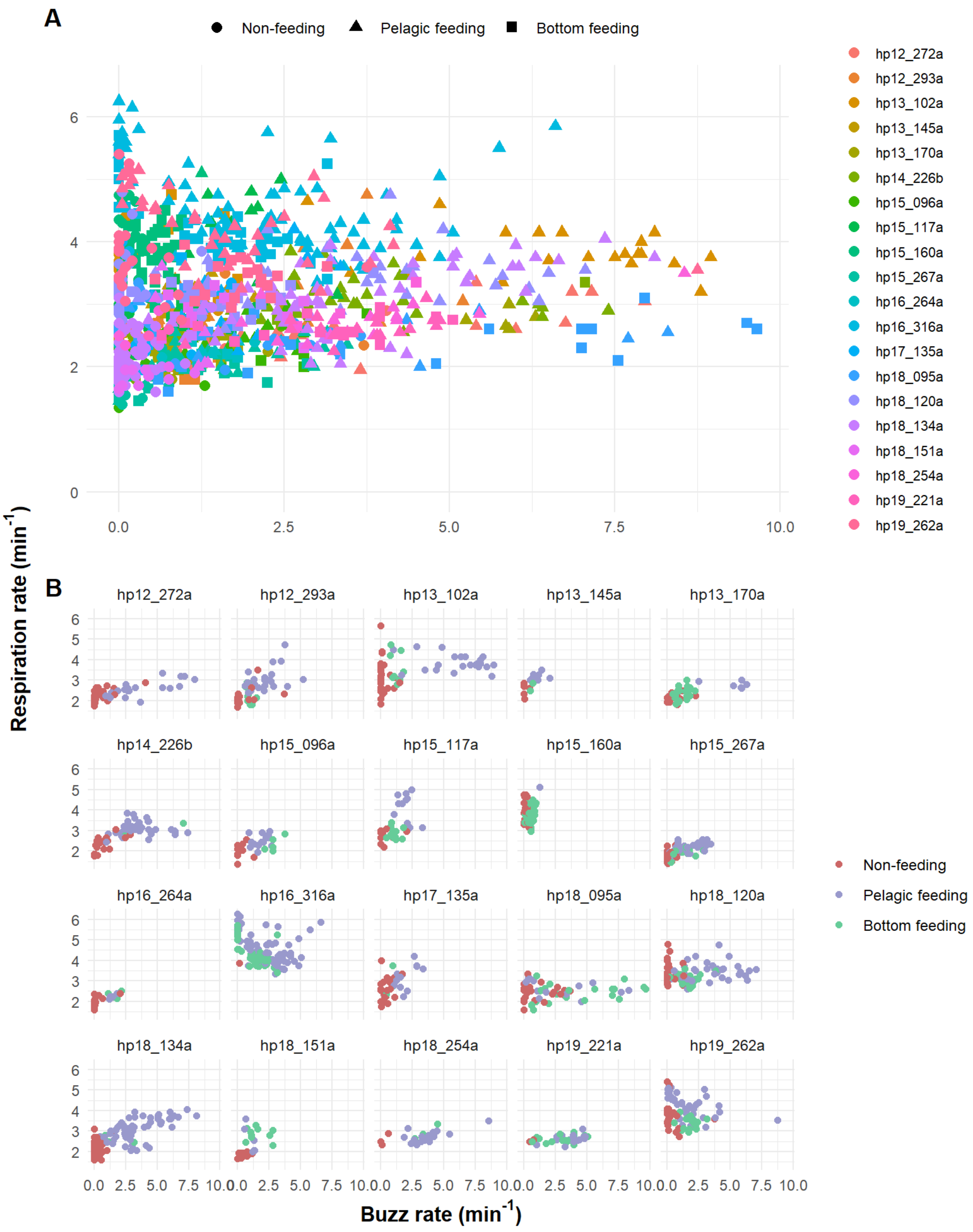

Fig. S6. Metabolic scope in harbor porpoises as a function of behavioral state. Relationship between respiration rate and buzz rate over 20-minute intervals. A) Observations are color-coded by ID and the shape relates to behavioral state. B) Dots are color-coded by behavioral state. Note that we chose to use 20-minute bins as they correspond to 10 times the 95th percentile of the dive cycle duration of the tagged porpoises.

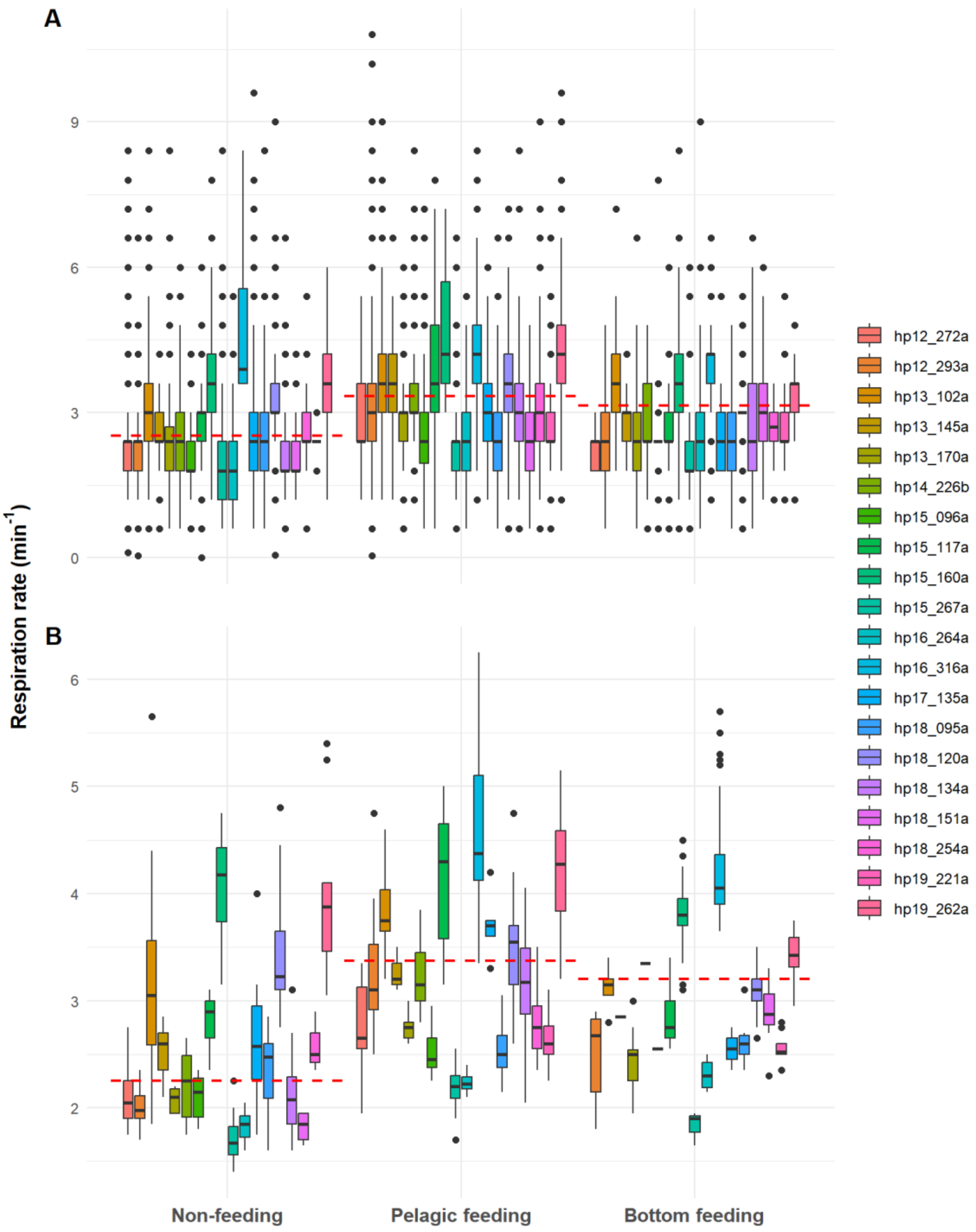

Fig. S7. Boxplots of respiration rates (min^{-1}) in A) individual dive cycles and B) 20-minute intervals as a function of behavioral state for the 20 free-ranging harbor porpoises. Each colored box represents one of the 20 tagged porpoises. Box limits are first and third quartile and whiskers are minimum and maximum values excluding outliers (smallest observation greater than or equal to lower hinge - 1.5 x inter-quartile range or largest observation less than or equal to upper hinge + 1.5 x inter-quartile range). Red dashed line represents the median by behavioral state. Note we chose to use 20-minute intervals as they correspond to 10 times the 95th percentile of the dive cycle duration of the tagged porpoises.

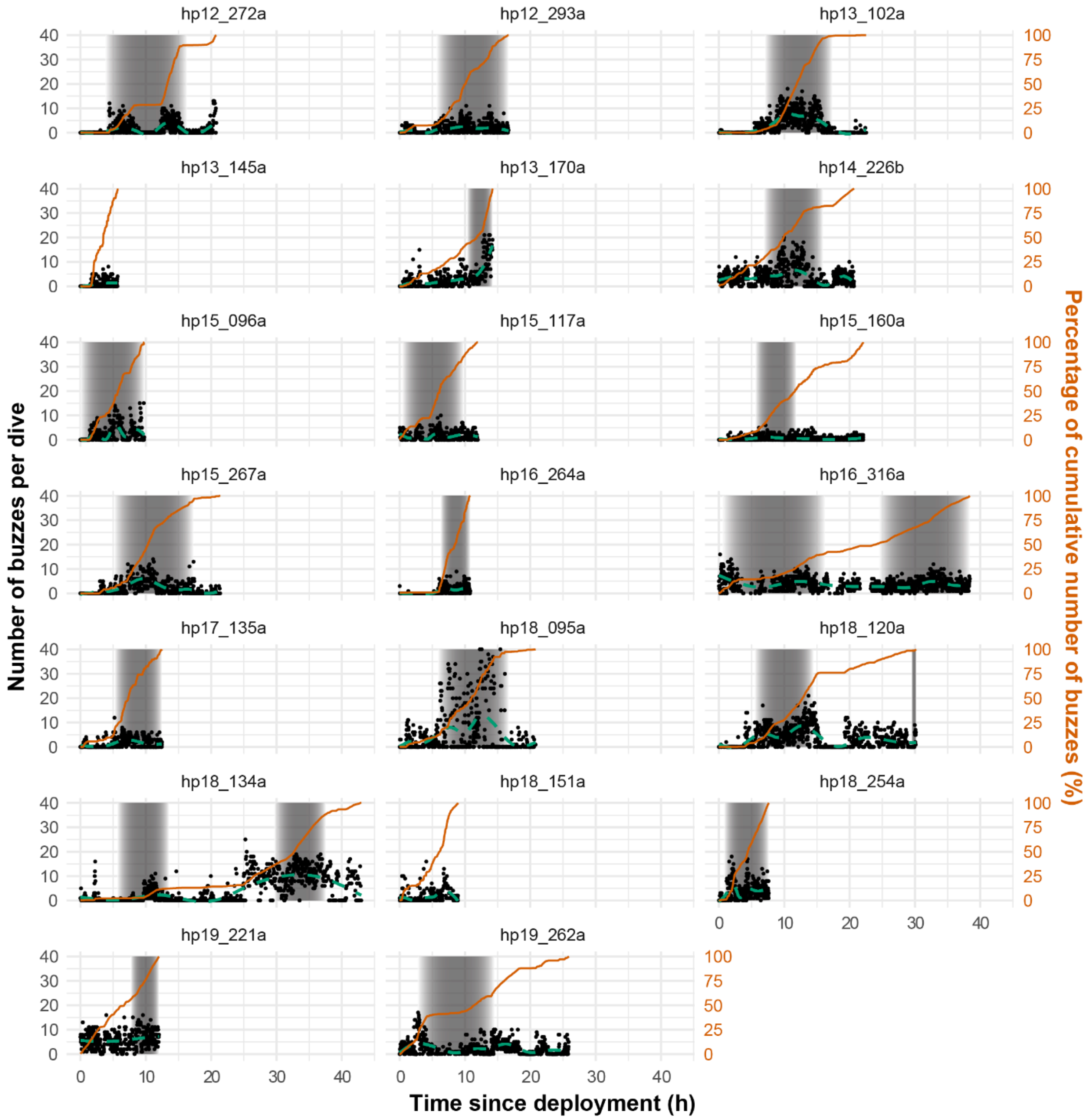

Fig. S8. Per-dive buzz count as a function of time since tag deployment for the tagged 20 harbor porpoises. The dashed green line is a Generalized additive model (GAM) with penalized cubic regression splines (i.e., default method in the *geom_smooth* function of the *ggplot2* package in R), and orange line shows the cumulative sum of number of buzzes over the deployment time expressed as percent proportion of the total number of buzzes. The shaded area represents nighttime.

Fig. S9. Overview of diving, feeding and breathing behavior of the 20 tagged harbor porpoises. Each graph represents one porpoise. (A) Dive profile of the harbor porpoise with prey-capture attempts (defined by echolocation buzzes) marked in red. (B) One-minute (dark red) and 20-minute (light red) buzz counts. C) Respiration rates averaged over 20-minute periods (orange) and 95th percentile of minimum specific acceleration (MSA, a proxy for activity) averaged over 5-second (green) and 20-minute periods (yellow). D) Energy balance calculated as the cumulative difference between the energy gained from prey captures and the metabolic energy expended (estimated via respirations). Starting from a null energy balance, 0 MJ, positive energy balance is depicted in green and negative in orange. The average energy turnover per respiration is calculated following Rojano-Doñate et al., (*13*), and the average energy per prey capture is calculated using the estimated field metabolic rate of the individual (calculated following Rojano-Doñate et al., (*13*)) divided by the total number of prey-capture attempts assuming a 90% assimilation efficiency. We assumed the calorific value of prey (i.e., fish) to be 4.2 kJ g^{-1} (*18*). The shaded area represents nighttime. Dashed lines in B and C represent incomplete 20-minute periods.

1) hp12_272a

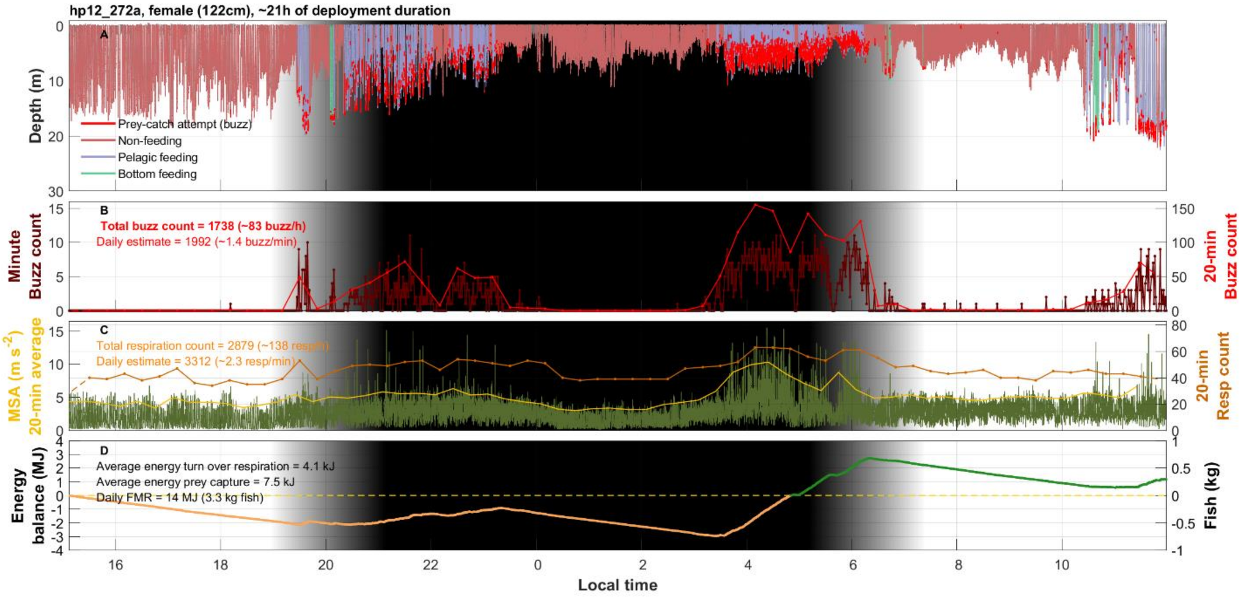

2) hp12_293a

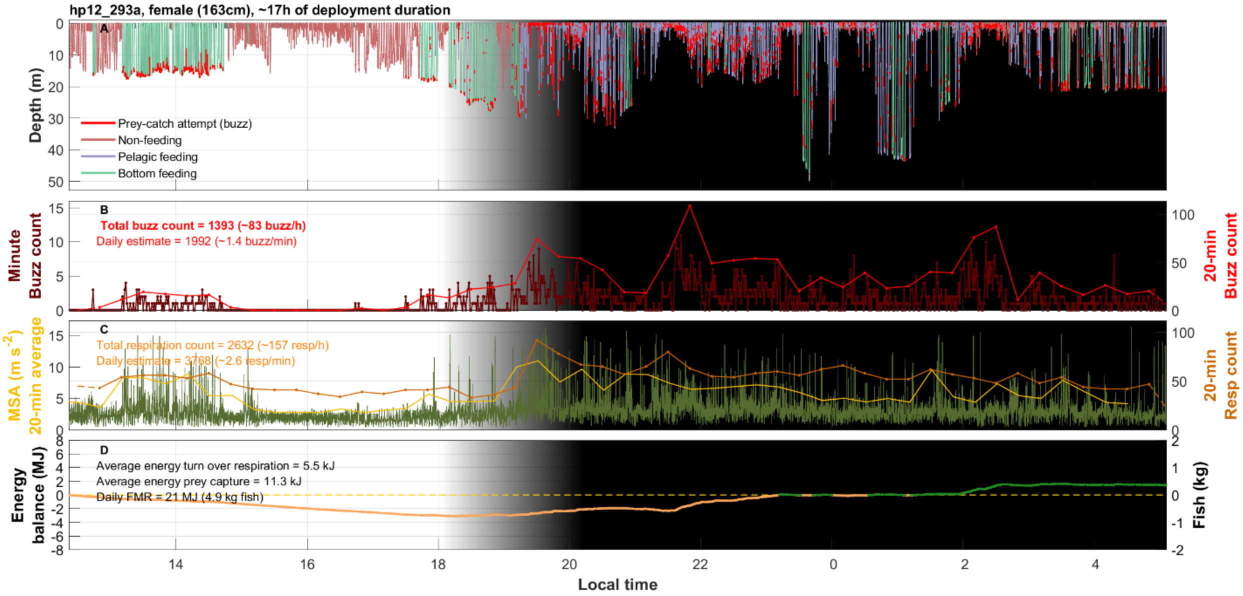

3) hp13_102a

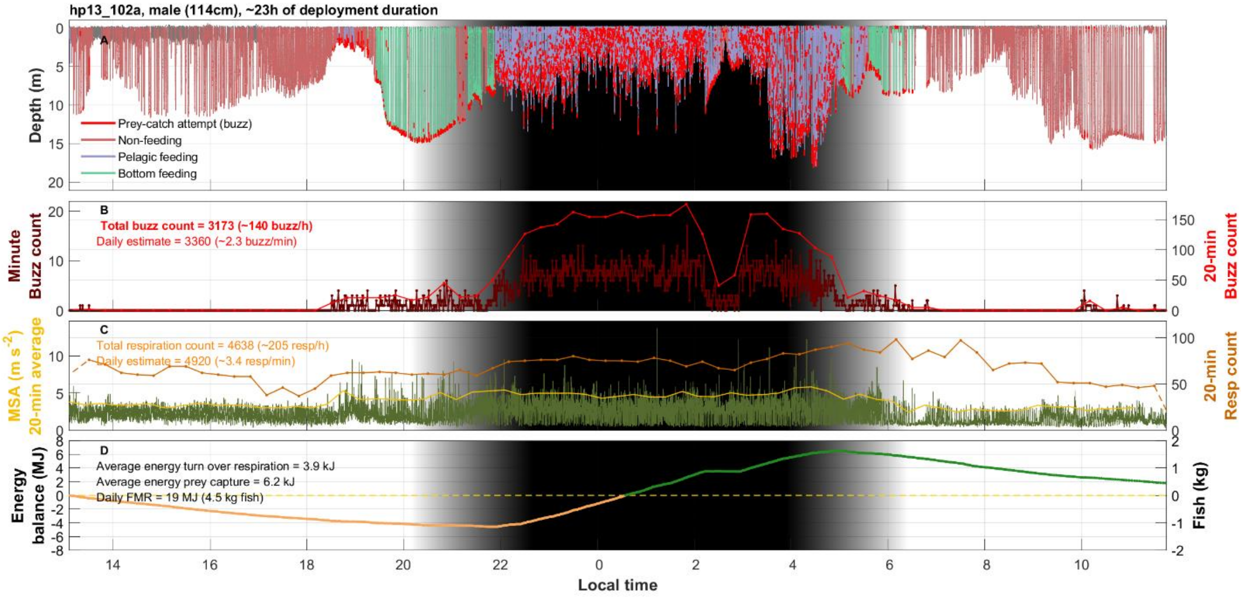

4) hp13_145a

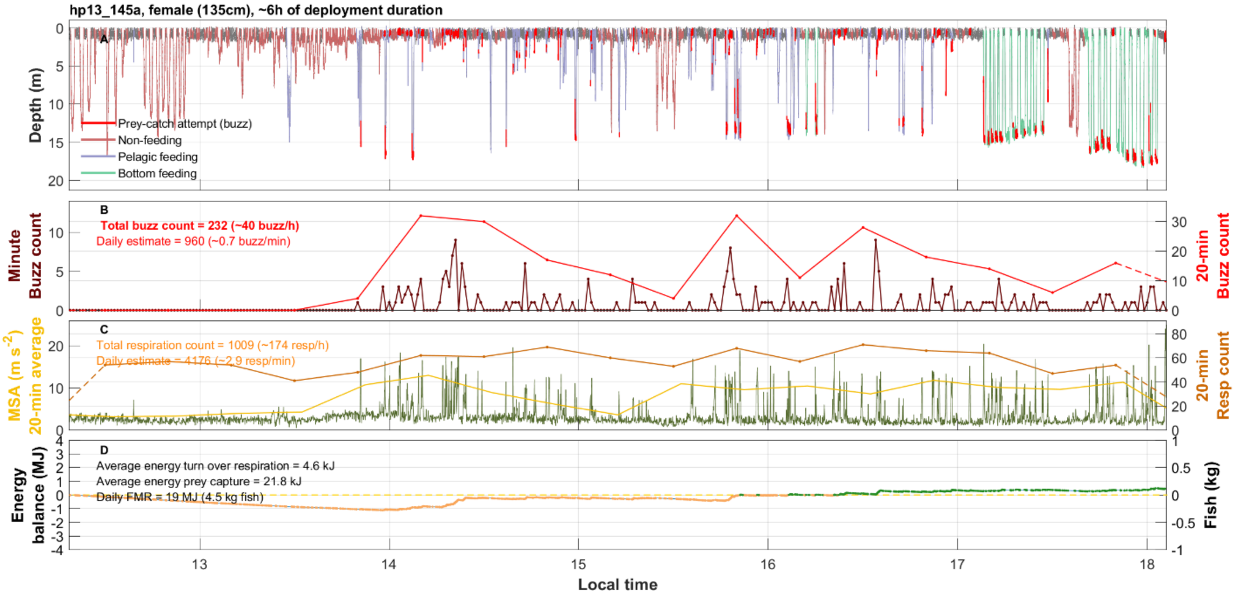

5) hp13_170a

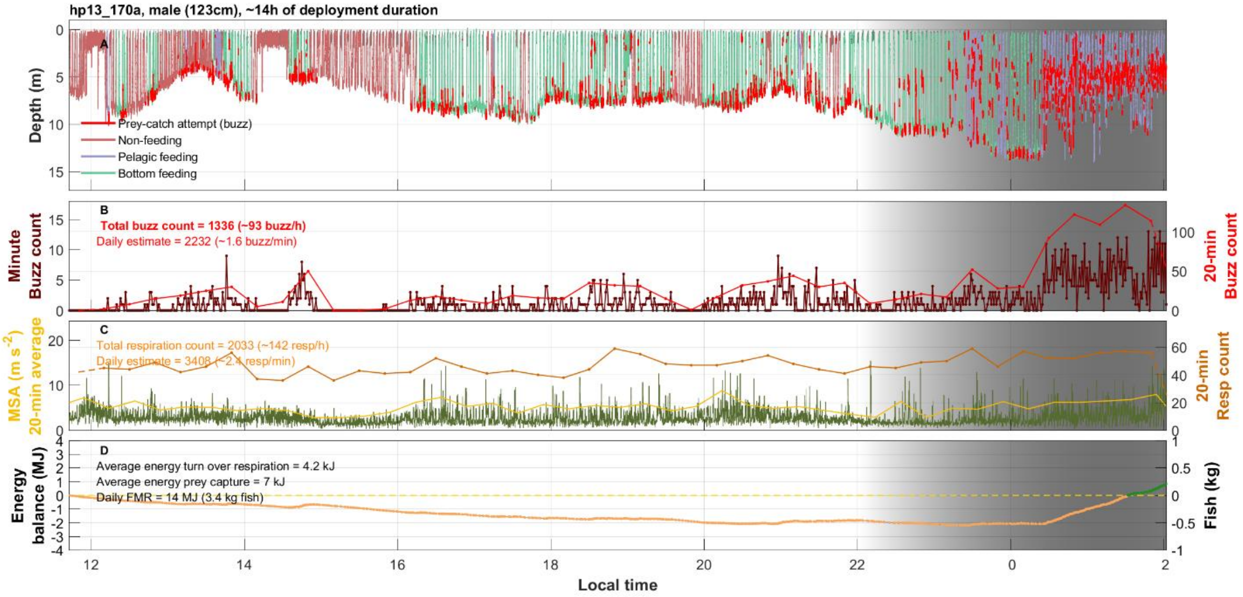

6) hp14_226b

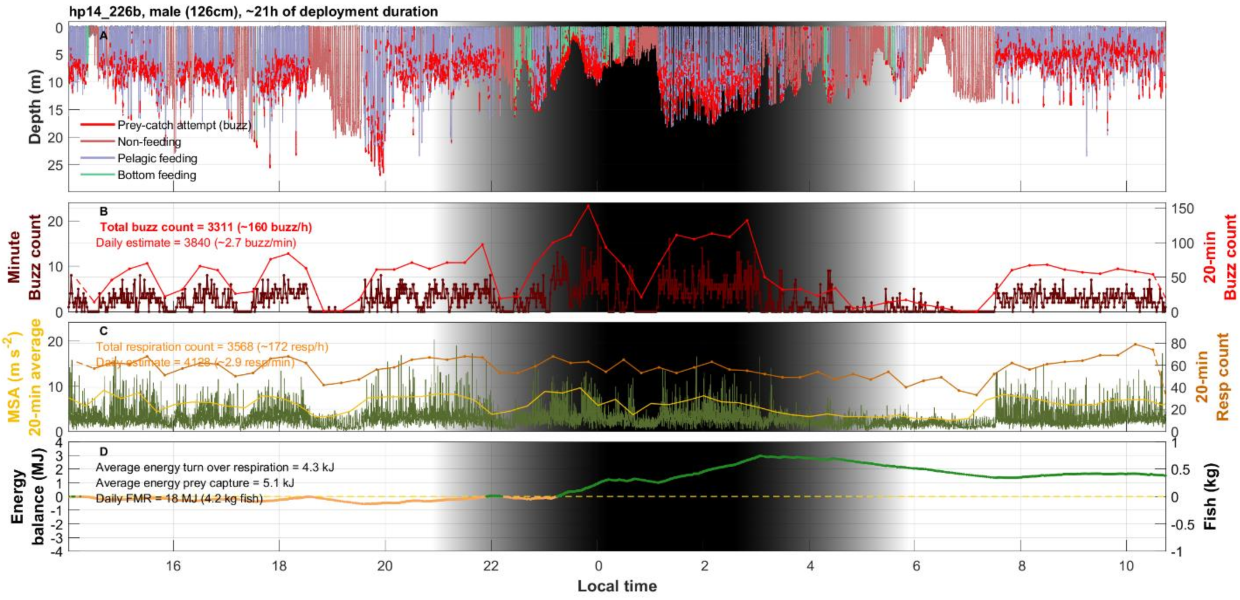

7) hp15_096a

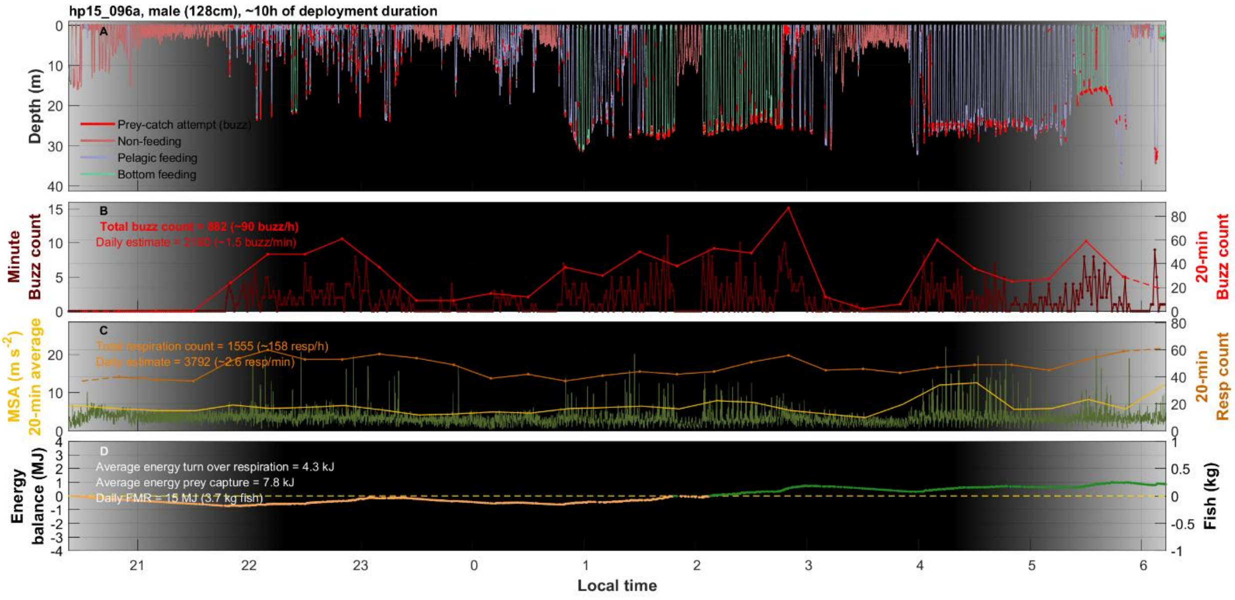

8) hp15_117a

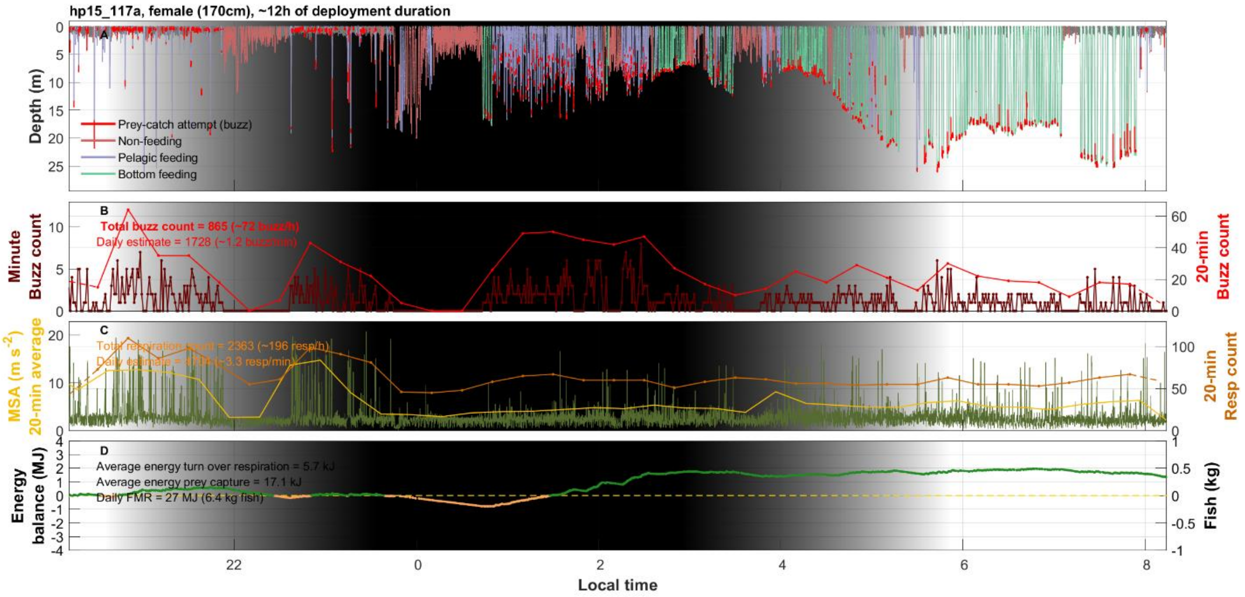

9) hp15_160a

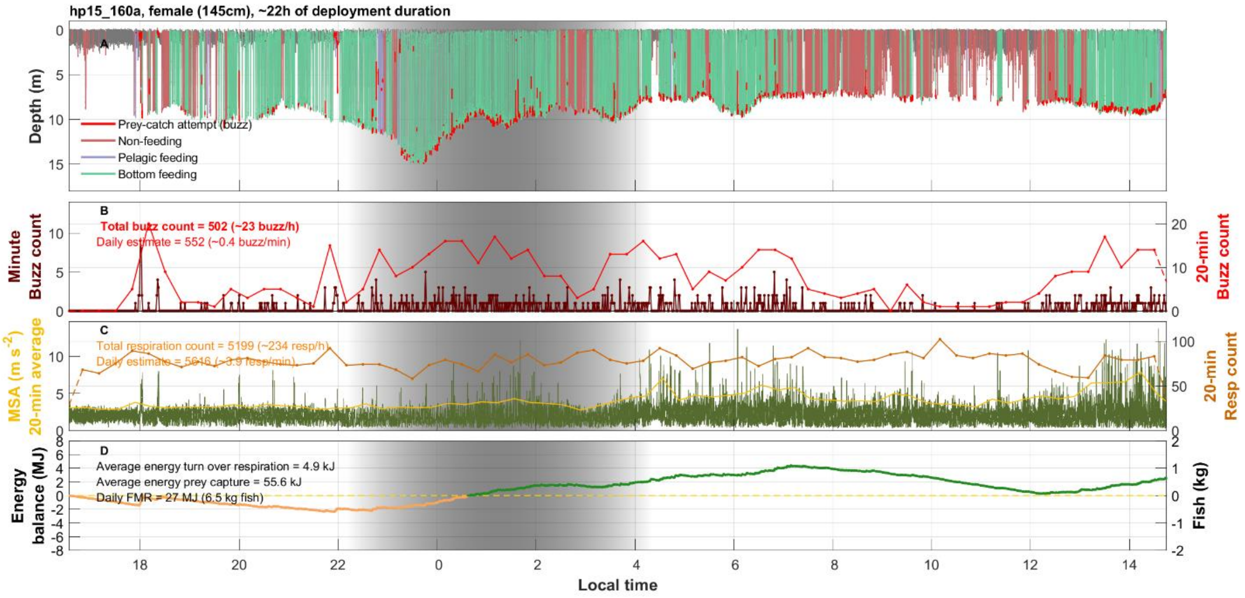

10) hp15_267a

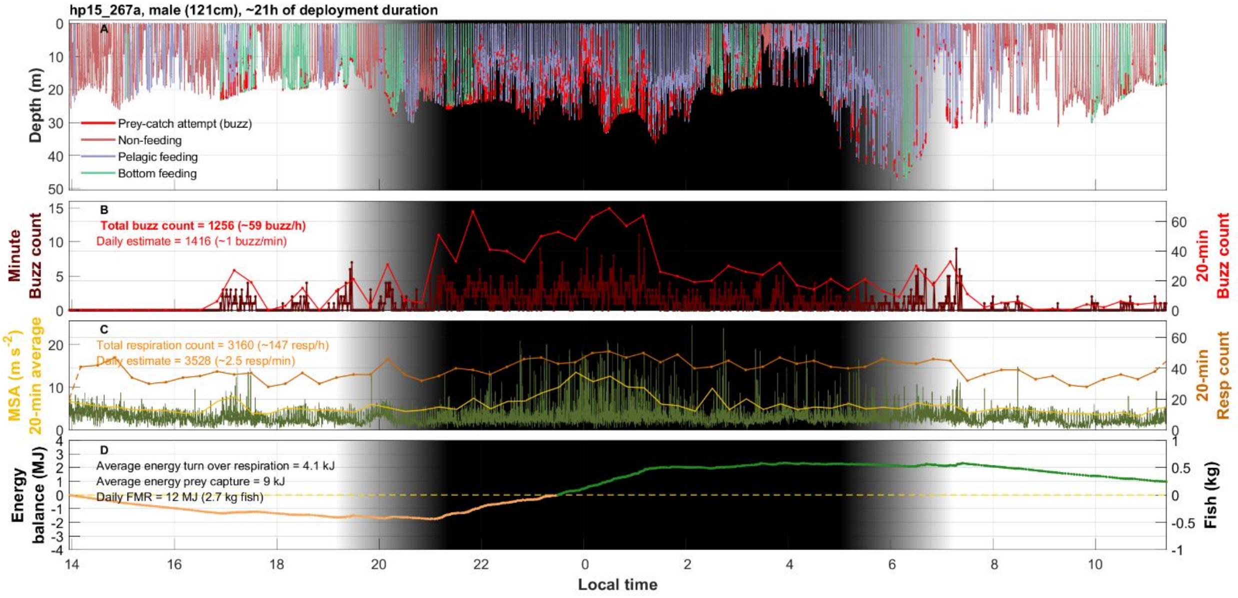

11) hp16_264a

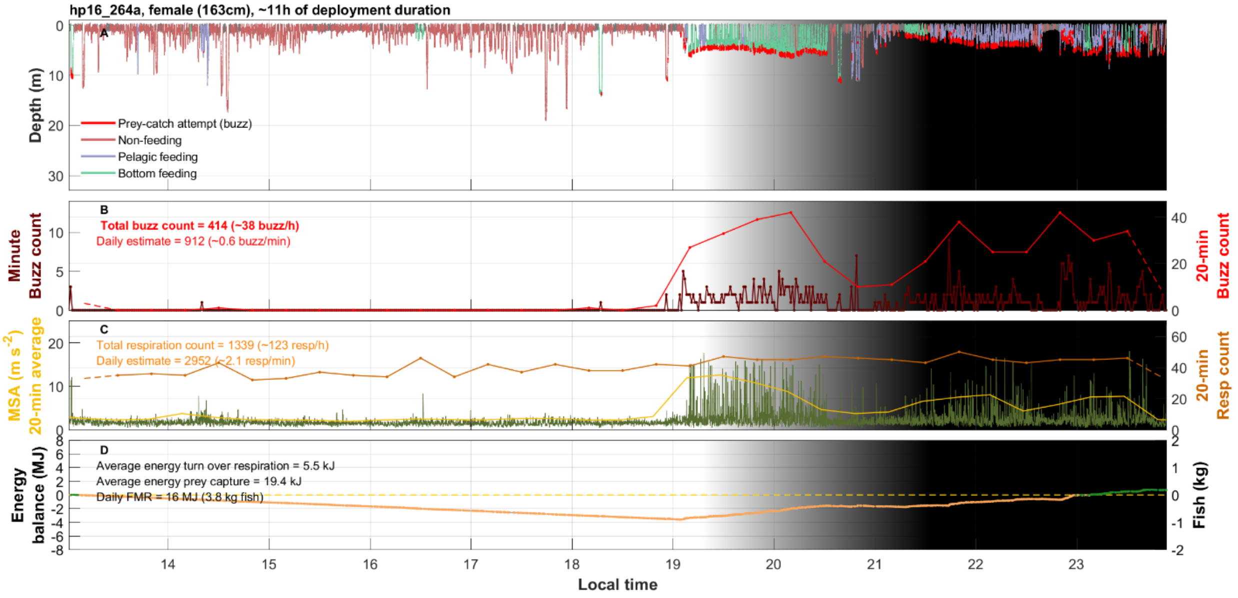

12) hp16_316a

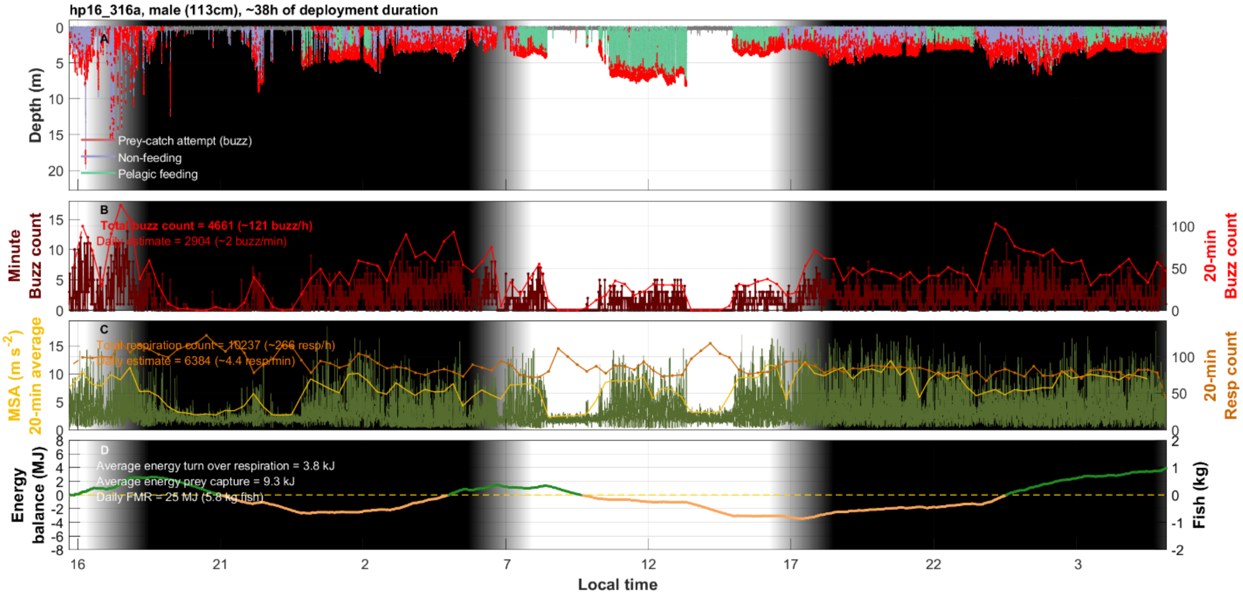

13) hp17_135a

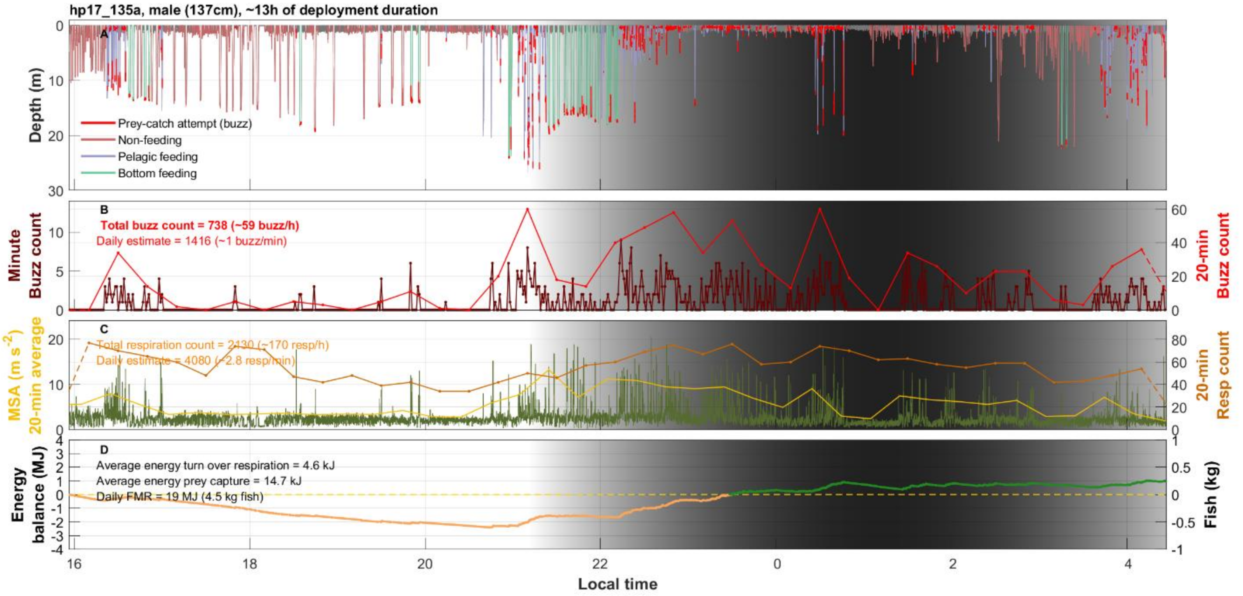

14) hp18_095a

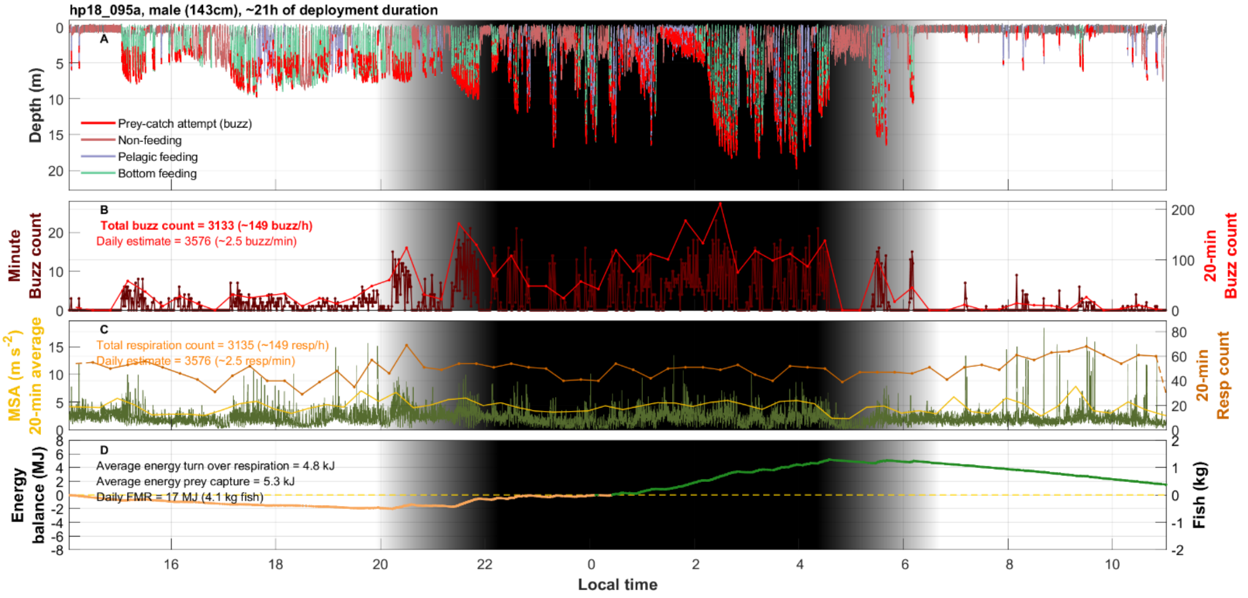

15) hp18_120a

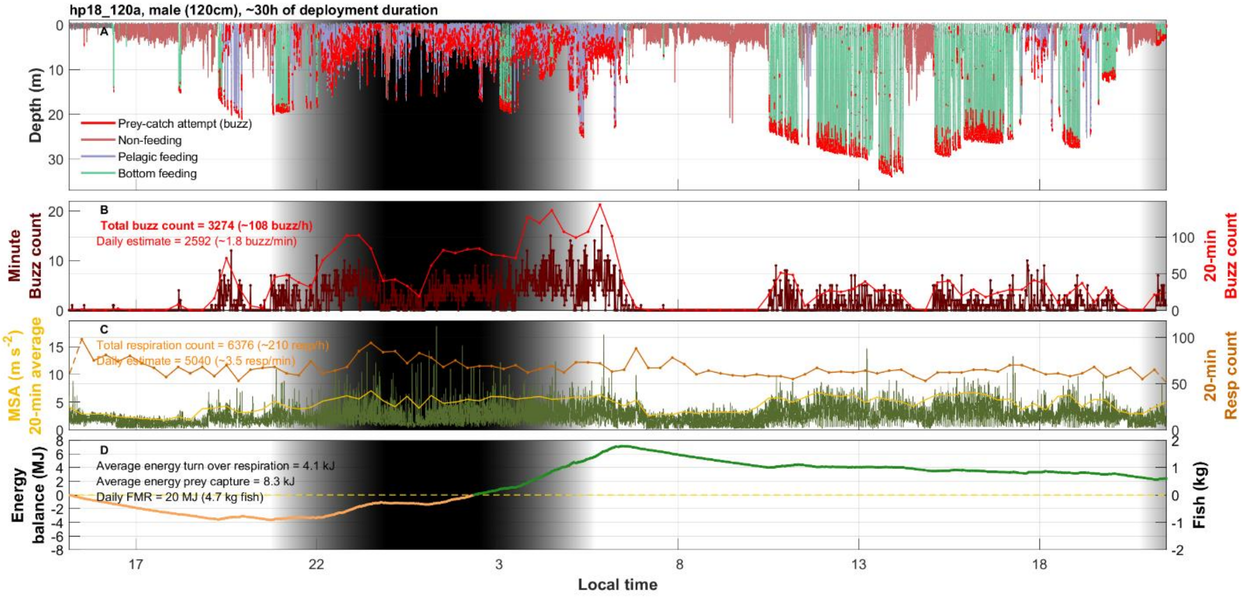

16) hp18_134a

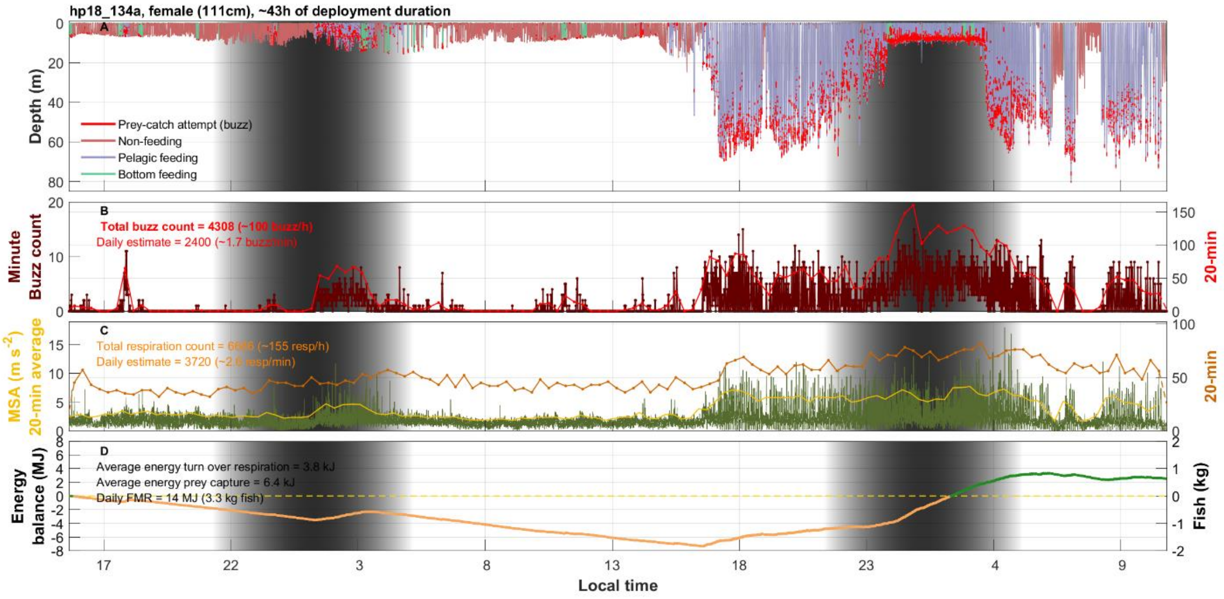

17) hp18_151a

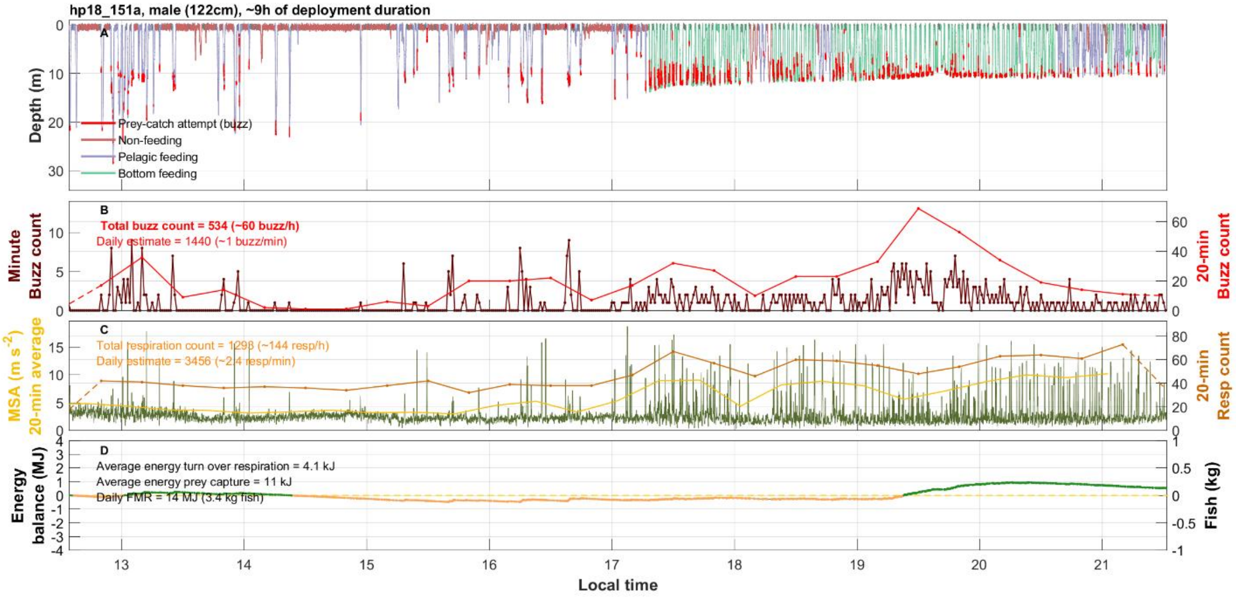

18) hp18_254a

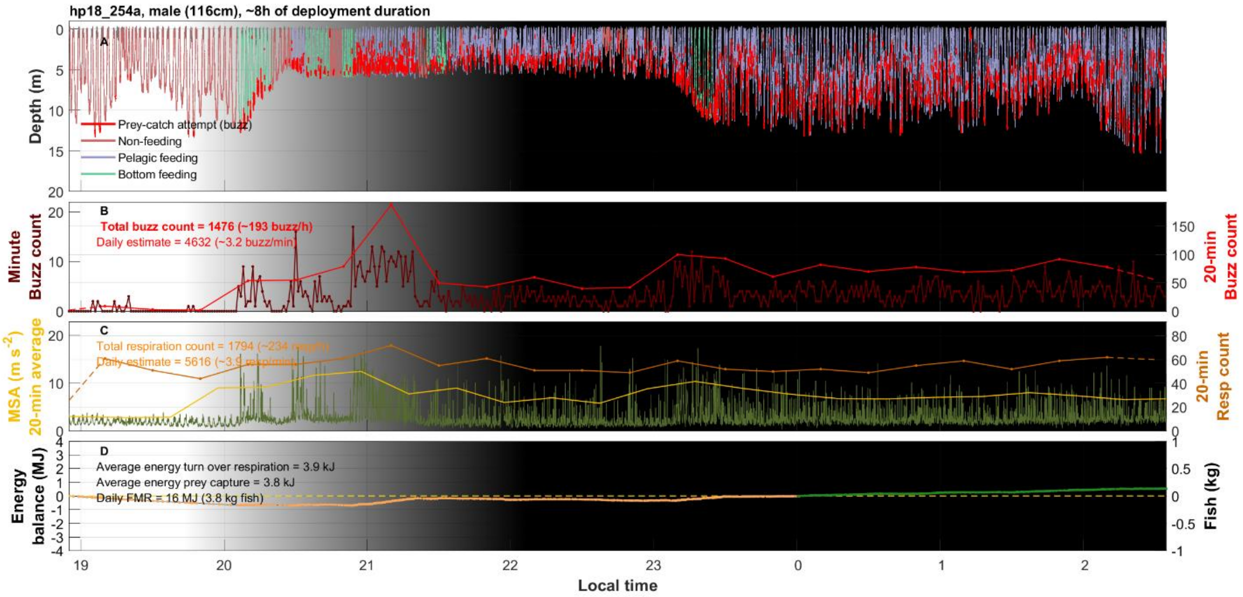

19) hp19_221a

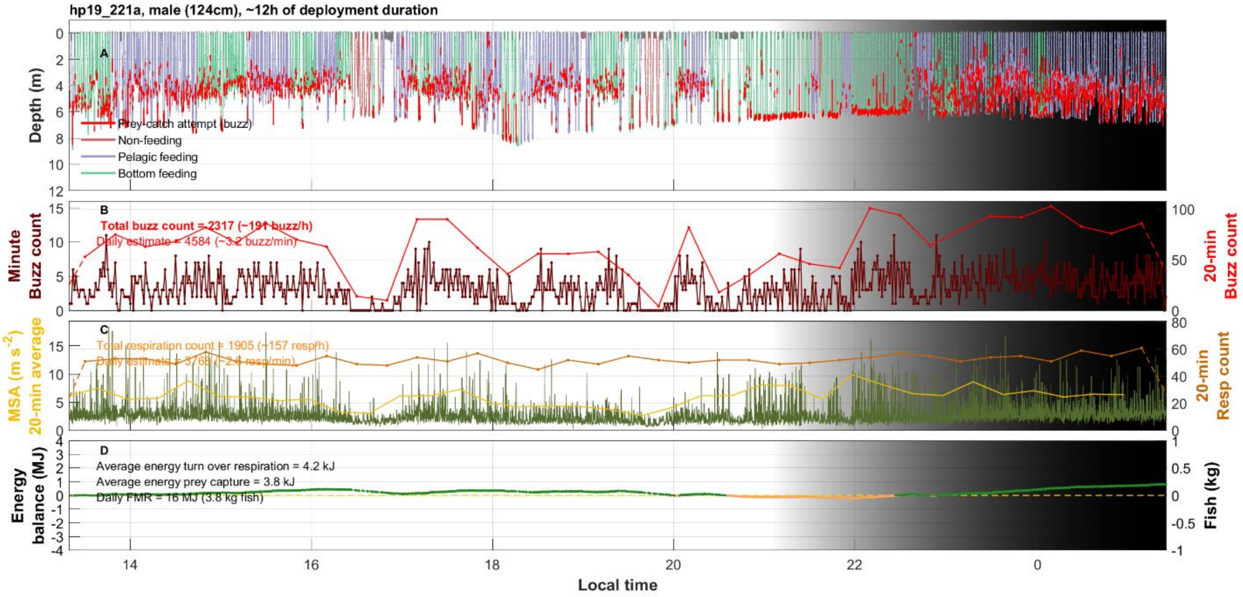

20) hp19_262a

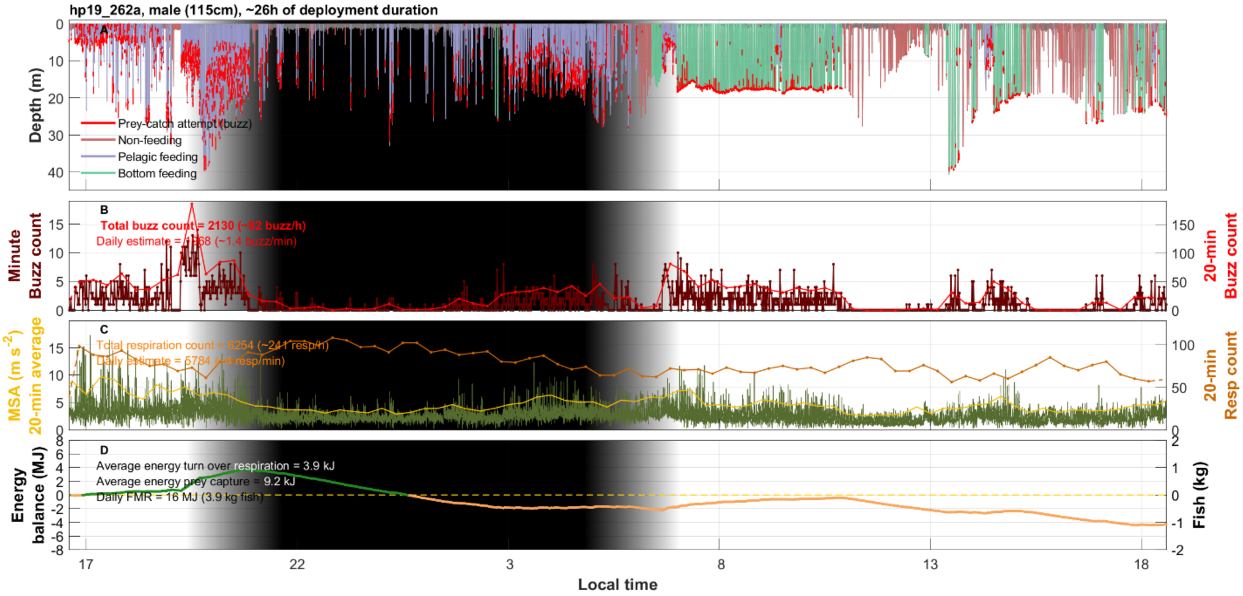

hp19_262a, male (115cm), ~26h of deployment duration
A
Depth (m)
Prey-catch attempt (buzz)
Non-feeding
Pelagic feeding
Bottom feeding
B
Minute Buzz count
Total buzz count = 2130 (~82 buzz/h)
20-min Buzz count
C
MSA (m s-2) 20-min average
Total respiration count = 6254 (~241 resp/h)
20-min Resp count
D
Energy balance (MJ)
Average energy turn over respiration = 3.9 kJ
Average energy prey capture = 9.2 kJ
Daily FMR = 16 MJ (3.9 kg fish)
Fish (kg)
Local time

Fig. S10. Illustration of the aspect of the auditing tool in MATLAB, showing a respiration and a buzz (i.e., bio-sonar based prey-capture attempt). In the top panel, relative sound amplitude, spectrogram in the middle panel, and depth profile in the bottom panel.

(A) Respiration

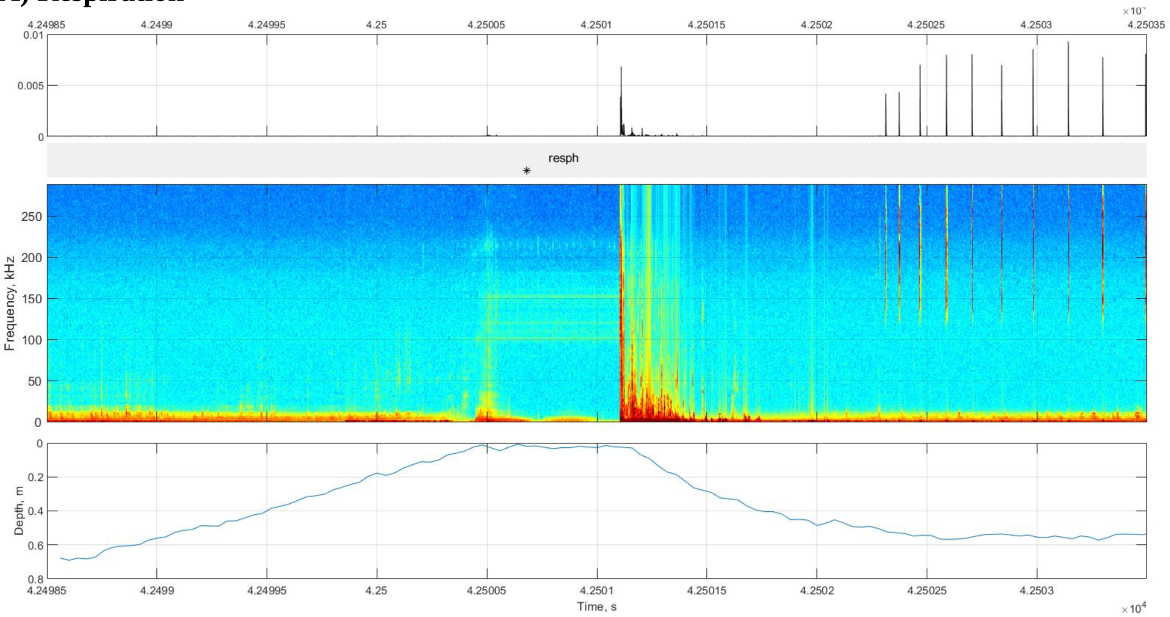

(B) Buzz

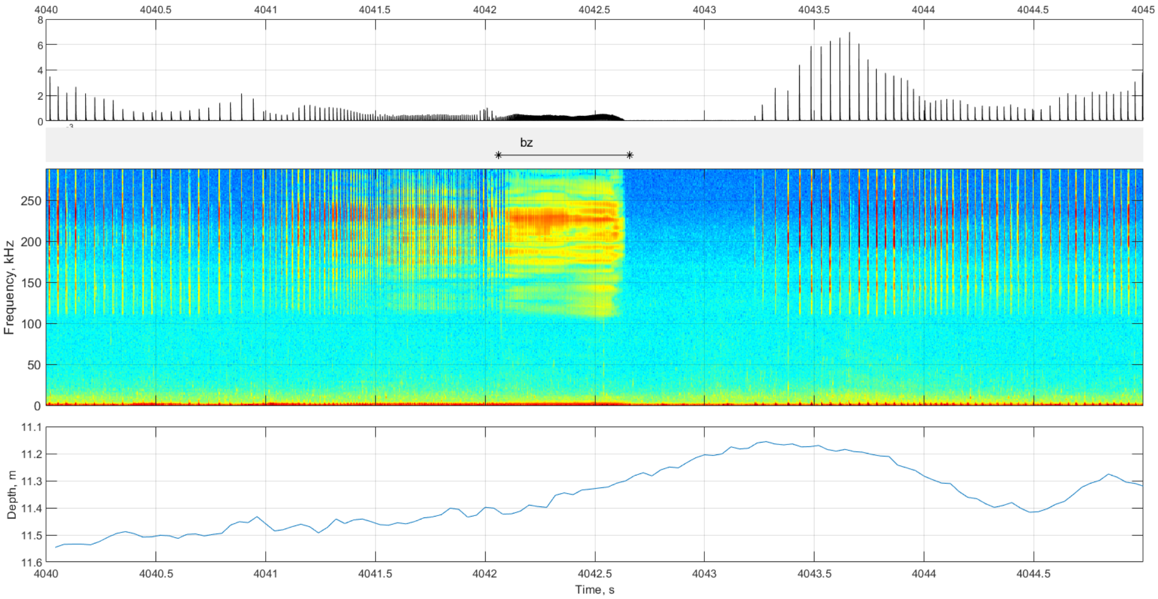

Table S1. Dive parameters of the 20 tagged harbor porpoises tagged in the Kattegat and Belt Seas. Total buzz count refers to the total buzz number of the complete deployment. Buzz rate (day^{-1}) is only calculated for deployments longer than 20 hours. The digits in the individuals' ID indicate the year and Julian day of tag deployment. Animals are sorted according to increasing standard body length. Note that total buzz counts differ slightly from Wisniewska et al., (*10*, *52*) because buzzes were reanalyzed to have a uniform buzz detection methodology across years. *accompanied by another porpoise, most likely its own calf. †potentially pregnant. ‡excluding first hour of deployment.

ID	Length (cm) - Sex (age)	Deployment duration (nighttime) (h)‡	Proportion of time				Max. dive depth (m)	Max. dive duration (min)	Total buzz count	Daytime buzz rate (h^{-1})	Nighttime buzz rate (h^{-1})	Buzz rate (day^{-1})
			Diving	Non-feeding	Pelagic feeding	Bottom feeding						
hp18_134a	111 ♀ (juvenile)	43.2 (15.8)	0.75	0.50	0.44	0.06	80	4.4	4242	59	167	2364
hp16_316a	113 ♂ (juvenile)	38.5 (29.5)	0.41	0.02	0.60	0.39	20	1.7	4300	65	126	2687
hp13_102a	114 ♂ (juvenile)	22.7 (10.3)	0.65	0.53	0.33	0.14	18	2.8	3046	11	283	3236
hp19_262a	115 ♂ (juvenile)	26.0 (14.2)	0.47	0.29	0.47	0.25	41	2.2	2037	77	80	1886
hp18_254a	116 ♂ (juvenile)	7.7 (0.8)	0.77	0.22	0.68	0.10	16	2.3	1450	16	209	-
hp18_120a	120 ♂ (juvenile)	30.3 (20.5)	0.61	0.38	0.34	0.27	34	4.5	3054	53	202	2427
hp15_267a	121 ♂ (juvenile)	21.4 (12.1)	0.83	0.34	0.48	0.18	48	3.3	1238	14	91	1389
hp12_272a	122 ♀ (juvenile)	20.9 (12.5)	0.8	0.72	0.27	0.01	23	2.1	1689	21	122	1948
hp18_151a	122 ♂ (juvenile)	9.0 (0)	0.68	0.37	0.27	0.36	29	2.0	530	59	-	-
hp13_170a	123 ♂ (juvenile)	14.3 (4.0)	0.77	0.31	0.16	0.54	14	3.6	1256	53	180	-
hp19_221a	124 ♂ (juvenile)	12.1 (4.4)	0.76	0.04	0.51	0.44	9	2.4	2297	166	235	-
hp14_226b	126 ♂ (juvenile)	20.7 (9.1)	0.72	0.32	0.61	0.06	27	2.5	3262	131	192	3784
hp15_096a	128 ♂ (juvenile)	9.8 (9.8)	0.72	0.35	0.50	0.15	38	2.4	804	-	82	-
hp13_145a	135 ♀ (juvenile)	5.8 (0)	0.56	0.49	0.38	0.13	18	2.1	208	36	-	-
hp17_135a	137 ♂ (adult)	12.5 (7.3)	0.48	0.55	0.35	0.10	27	1.7	598	17	70	-
hp18_095a	143 ♂ (adult)	21 (10.8)	0.56	0.43	0.24	0.32	20	3.3	3083	44	243	3542
hp15_160a†	145 ♀ (adult)	22.2 (6.3)	0.54	0.35	0.03	0.62	15	2.0	458	16	31	497
hp12_293a*	163 ♀ (adult)	16.7 (11.1)	0.66	0.37	0.40	0.23	50	2.7	1131	19	93	-
hp16_264a*	163 ♀ (adult)	10.9 (4.6)	0.67	0.62	0.19	0.19	19	2.2	403	5	81	-
hp15_117a†	170 ♀ (adult)	12 (9.3)	0.54	0.27	0.40	0.33	27	3.6	730	50	64	-
**MEAN**	**131**	**18.9**	**0.65**	**0.37**	**0.38**	**0.24**	**29**	**2.7**	**1791**	**48**	**142**	**2376**

Before\After	Non-feeding	Pelagic feeding	Bottom feeding
**Non-feeding**	0.89	0.07	0.04
**Pelagic feeding**	0.07	0.90	0.03
**Bottom feeding**	0.09	0.07	0.84

Table S2. Transition probabilities between behavioral states of the most parsimonious Hidden Markov Model (HMM) for categorizing dive types of harbor porpoises in the Kattegat and Belt seas.

REFERENCES AND NOTES

1. C . Carbone, G. M. Mace, S. C. Roberts, D. W. Macdonald, Energetic constraints on the diet of terrestrial carnivores. Nature 402, 286–288 (1999).

2. C . Carbone, A. Teacher, J. M. Rowcliffe, The costs of carnivory. PLoS Biol. 5, e22 (2007).

3. T . M. Williams, M. Peter-Heide Jørgensen, A. M. Pagano, C. M. Bryce, Hunters versus hunted: New perspectives on the energetic costs of survival at the top of the food chain. Funct. Ecol. 34, 2015–2029 (2020).

4. J. A. Goldbogen, D. E. Cade, D. M. Wisniewska, J. Potvin, P. S. Segre, M. S. Savoca, E. L. Hazen, M. F. Czapanskiy, S. R. Kahane-Rapport, S. L. DeRuiter, S. Gero, P. Tønnesen, W. T. Gough, M. B. Hanson, M. M. Holt, F. H. Jensen, M. Simon, A. K. Stimpert, P. Arranz, D. W. Johnston, D. P. Nowacek, S. E. Parks, F. Visser, A. S. Friedlaender, P. L. Tyack, P. T. Madsen, N. D. Pyenson, Why whales are big but not bigger: Physiological drivers and ecological limits in the age of ocean giants. Science 366, 1367–1372 (2019).

5. M. A. Tucker, T. L. Rogers, Examining predator-prey body size, trophic level and body mass across marine and terrestrial mammals. Proc. R. Soc. B Biol. Sci. 281, 2103 (2014).

6. D. L. Kramer, The behavioral ecology of air breathing by aquatic animals. Can. J. Zool. 66, 89–94 (1988).

7. A. I. Houston, Assessing models of optimal diving. Trends Ecol. Evol. 26, 292–297 (2011).

8. J. A. Goldbogen, D. E. Cade, J. Calambokidis, A. S. Friedlaender, J. Potvin, P. S. Segre, A. J. Werth, How Baleen Whales feed: The biomechanics of engulfment and filtration. Ann. Rev. Mar. Sci. 9, 367–386 (2017).

9. J. A. Blakeway, J. P. Y. Arnould, A. J. Hoskins, P. Martin-Cabrera, G. J. Sutton, L. A. Huckstadt, D. P. Costa, D. Páez-Rosas, S. Villegas-Amtmann, Influence of hunting strategy on foraging efficiency in Galapagos sea lions. PeerJ. 9, 11206 (2021).

10. D. M. Wisniewska, M. Johnson, J. Teilmann, L. Rojano-Doñate, J. Shearer, S. Sveegaard, L. A. Miller, U. Siebert, P. T. Madsen, Ultra-high foraging rates of harbor porpoises make them vulnerable to anthropogenic disturbance. Curr. Biol. 26, 1–6 (2016).

11. Y. Naito, D. P. Costa, T. Adachi, P. W. Robinson, M. Fowler, A. Takahashi, Unravelling the

mysteries of a mesopelagic diet: A large apex predator specializes on small prey. Funct. Ecol. 27, 710–717 (2013).

12. P. T. Madsen, N. A. de Soto, P. Arranz, M. Johnson, Echolocation in Blainville's beaked whales (Mesoplodon densirostris). J. Comp. Physiol. A Neuroethol. Sensory, Neural, Behav. Physiol. 199, 451–469 (2013).

13. L . Rojano-Doñate, B. I. McDonald, D. M. Wisniewska, M. Johnson, J. Teilmann, M. Wahlberg, J. Højer-Kristensen, P. T. Madsen, High field metabolic rates of wild harbour porpoises. J. Exp. Biol. 221, jeb185827 (2018).

14. B. K. Ahlborn, R. W. Blake, Lower size limit of aquatic mammals. Am. J. Phys. 67, 920–922 (1999).

15. J. A. Goldbogen, P. T. Madsen, The evolution of foraging capacity and gigantism in cetaceans. J. Exp. Biol. 221, jeb166033 (2018).

16. M. Johnson, N. Aguilar de Soto, P. T. Madsen, Studying the behaviour and sensory ecology of marine mammals using acoustic recording tags: A review. Mar. Ecol. Process Ser. 395, 55–73 (2009).

17. C . Lockyer, C. Kinze, Status, ecology and life history of harbour porpoise (Phocoena phocoena), in Danish waters. NAMMCO Sci. Publ. 5, 143 (2014).

18. C . G. Booth, M. Guilpin, A.-K. Darias-O'Hara, J. M. Ransijn, M. Ryder, D. Rosen, E. Pirotta, S. Smout, E. A. McHuron, J. Nabe-Nielsen, D. P. Costa, Estimating energetic intake for marine mammal bioenergetic models. Conserv. Physiol. 11, 1–22 (2023).

19. A. J. Wright, T. Akamatsu, K. N. Mouritsen, S. Sveegaard, R. Dietz, J. Teilmann, Silent porpoise: Potential sleeping behaviour identified in wild harbour porpoises. Anim. Behav. 133, 211–222 (2017).

20. T . Schaffeld, S. Bräger, A. Gallus, M. Dähne, K. Krügel, A. Herrmann, M. Jabbusch, T. Ruf, U. Verfuß, H. Benke, J. Koblitz, Diel and seasonal patterns in acoustic presence and foraging behaviour of free-ranging harbour porpoises. Mar. Ecol. Prog. Ser. 547, 257–272 (2016).

21. J. Stedt, M. Wahlberg, J. Carlström, P. Nilsson, M. Amundin, N. Oskolkov, P. Carlsson, Micro-scale spatial preference and temporal cyclicity linked to foraging in harbour

porpoises. Mar. Ecol. Prog. Ser. 708, 143–161 (2023).
22. Y. Y. Watanabe, E. A. Baranov, N. Miyazaki, Ultrahigh foraging rates of Baikal seals make tiny endemic amphipods profitable in Lake Baikal. Proc. Natl. Acad. Sci. U.S.A. 117, 31242–31248 (2020).
23. T . Adachi, A. Takahashi, D. P. Costa, P. W. Robinson, L. A. Hückstädt, S. H. Peterson, R. R. Holser, R. S. Beltran, T. R. Keates, Y. Naito, Forced into an ecological corner: Round-the- clock deep foraging on small prey by elephant seals. Sci. Adv. 7, 3628–3640 (2021).
24. L . C. Yeates, T. M. Williams, T. L. Fink, Diving and foraging energetics of the smallest marine mammal, the sea otter (Enhydra lutris). J. Exp. Biol. 210, 1960–1970 (2007).
25. G. G. Esslinger, J. L. Bodkin, A. R. Breton, J. M. Burns, D. H. Monson, Temporal patterns in the foraging behavior of sea otters in Alaska. J. Wildl. Manage. 78, 689–700 (2014).
26. A. Fahlman, M. Brodsky, S. Miedler, S. Dennison, M. Ivanc, G. Levine, J. Rocho-levine, M. Manley, J. Rocabert, A. Borque-espinosa, Ventilation and gas exchange before and after voluntary static surface breath-holds in clinically healthy bottlenose dolphins, Tursiops truncatus. J. Exp. Biol. 222, jeb192211 (2019).
27. J. Z. Reed, C. Chambers, C. J. Hunter, C. Lockyer, R. Kastelein, M. A. Fedak, R. G. Boutilier, O. Paper, J. Z. Reed, C. Chambers, C. J. Hunter, C. Lockyer, R. Kastelein, M. A. Fedak, R. G. Boutilier, Gas exchange and heart rate in the harbour porpoise. J. Comp. Physiol. -B Biochem. Syst. Environ. Physiol. 170, 1–10 (2000).
28. G. L. Kooyman, D. H. Kerem, W. B. Campbell, J. J. Wright, Pulmonary function in freely diving Weddell seals, Leptonychotes weddelli. Respir. Physiol. 12, 271–282 (1971).
29. C . E. Sparling, J. Y. Georges, S. L. Gallon, M. Fedak, D. Thompson, How long does a dive last? Foraging decisions by breath-hold divers in a patchy environment: A test of a simple model. Anim. Behav. 74, 207–218 (2007).
30. B. I. McDonald, S. L. Elmegaard, M. Johnson, D. M. Wisniewska, L. Rojano-Doñate, A. Galatius, U. Siebert, J. Teilmann, P. T. Madsen, High heart rates in hunting harbour porpoises. Proc. R. Soc. B Biol. Sci. 288, 1596 (2021).
31. R. A. Kastelein, S. H. Nieuwstraten, M. W. A. Verstegen, "Passage Time of Carmine Red Dye

through the Digestion Tract" in The biology of the harbour porpoise, A. J. Read, P. R. Wiepkema, P. E. Nachtigall, Eds. (De Spil Publishers, Woerden, The Netherlands, 1997), pp. 265–275.

32. M. Diaz Gomez, D. A. S. Rosen, A. W. Trites, Net energy gained by northern fur seals (Callorhinus ursinus) is impacted more by diet quality than by diet diversity. Can. J. Zool. 94, 123–135 (2016).

33. S. K. A. Videsen, M. Simon, F. Christiansen, A. Friedlaender, J. Goldbogen, H. Malte, P. Segre, T. Wang, M. Johnson, P. T. Madsen, Cheap gulp foraging of a giga-predator enables efficient exploitation of sparse prey. Sci. Adv. 9, eade3889 (2023).

34. T . Jeanniard-du-Dot, A. W. Trites, J. P. Y. Arnould, J. R. Speakman, C. Guinet, Activity-specific metabolic rates for diving, transiting, and resting at sea can be estimated from time–activity budgets in free-ranging marine mammals. Ecol. Evol. 7, 2969–2976 (2017).

35. T . Jeanniard-du-Dot, C. Guinet, J. P. Y. Arnould, J. R. Speakman, A. W. Trites, Accelerometers can measure total and activity-specific energy expenditures in free-ranging marine mammals only if linked to time-activity budgets. Funct. Ecol. 31, 377–386 (2017).

36. T . M. Williams, L. A. Fuiman, M. Horning, R. W. Davis, The cost of foraging by a marine predator, the Weddell seal Leptonychotes weddellii: Pricing by the stroke. J. Exp. Biol. 207, 973–982 (2004).

37. M. L. Gorman, M. G. Mills, J. P. Raath, J. R. Speakman, High hunting costs make African wild dogs vulnerable to kleptoparasitism by hyaenas. Nature 391, 479–481 (1998).

38. T . M. Williams, L. Yeates, The energetics of foraging in large mammals: A comparison of marine and terrestrial predators. Int. Congr. Ser. 1275, 351–358 (2004).

39. A. Werth, Feeding in Marine Mammals. In: Feeding, 487–526. Academic Press (2000).

40. H . Vance, P. T. Madsen, N. Aguilar de Soto, D. M. Wisniewska, M. Ladegaard, S. Hooker, M. Johnson, Echolocating toothed whales use ultra-fast echo-kinetic responses to track evasive prey. eLife 10, e68825 (2021).

41. A. I. Houston, Energetic constraints and foraging efficiency. Behav. Ecol. 6, 393–396 (1995).

42. Y. Tremblay, Y. Cherel, Benthic and pelagic dives: A new foraging behaviour in rockhopper penguins. Mar. Ecol. Prog. Ser. 204, 257–267 (2000).

43. B. L. Chilvers, I. S. Wilkinson, Diverse foraging strategies in lactating New Zealand sea lions. Mar. Ecol. Prog. Ser. 378, 299–308 (2009).

44. T . M. Williams, The evolution of cost efficient swimming in marine mammals: Limits to energetic optimization. Philos. Trans. R. Soc. B Biol. Sci. 354, 193–201 (1999).

45. R. W. Davis, Marine Mammals. Adaptations for an Aquatic Life (Springer Nature Switzerland AG, 2019).

46. E . Pirotta, C. G. Booth, D. P. Costa, E. Fleishman, S. D. Kraus, D. Lusseau, D. Moretti, L. F. New, R. S. Schick, L. K. Schwarz, S. E. Simmons, L. Thomas, P. L. Tyack, M. J. Weise, R. S. Wells, J. Harwood, Understanding the population consequences of disturbance. Ecol. Evol. 8, 9934–9946 (2018).

47. ICE S, "Cod (Gadus morhua) in Subdivision 21 (Kattegat). In Report of the ICES Advisory Committee, 2019. ICES Advice 2019, cod.27.21, https://doi.org/10.17895/ices.advice.4745" (2019).

48. M. Obst, S. Vicario, K. Lundin, M. Berggren, A. Karlsson, R. Haines, A. Williams, C. Goble, C. Mathew, A. Güntsch, Marine long-term biodiversity assessment suggests loss of rare species in the Skagerrak and Kattegat region. Mar. Biodivers. 48, 2165–2176 (2018).

49. C . R. Elliser, S. Hessing, K. H. Maciver, M. A. Webber, W. Keener, Harbor porpoises (Phocoena phocoena vomerina) catching and handling large fish on the U.S. West Coast. Aquat. Mamm. 46, 191–199 (2020).

50. D. M. Wisniewska, M. Johnson, J. Teilmann, U. Siebert, A. Galatius, R. Dietz, P. T. Madsen, High rates of vessel noise disrupt foraging in wild harbour porpoises (Phocoena phocoena). Proc. R. Soc. B Biol. Sci. 285, 20172314 (2018).

51. P. Snoeijs-Leijonmalm, H. Schubert, T. Radziejewska, Biological Oceanography of the Baltic Sea (2017).

52. J. Teilmann, F. Larsen, G. G. Desportes, Time allocation and diving behaviour of harbour porpoises (Phocoena phocoena) in Danish and adjacent waters. J. Cetacean Res. Manag. 9, 201–210 (2007).

53. M. P. Johnson, P. L. Tyack, A digital acoustic recording tag for measuring the response of wild marine mammals to sound. IEEE J. Ocean. Eng. 28, 3–12 (2003).

54. D. M. Wisniewska, M. Johnson, K. Beedholm, P. T. Madsen, Adaptive prey tracking by echolocating porpoises studied with acoustic tags. J. Acoust. Soc. Am. 131, 3523–3523 (2012).

55. S. L. DeRuiter, A. Bahr, M.-A. Blanchet, S. Fobian Hansen, J. Højer Kristensen, P. T. Madsen, M. Wahlberg, Acoustic behaviour of echolocating porpoises during prey capture. J. Exp. Biol. 212, 3100–3107 (2009).

56. L . Scrucca, M. Fop, T. B. Murphy, A. Raftery, mclust 5: Clustering, classification and density estimation using Gaussian finite mixture models. R J. 8, 289–317 (2016).

57. R Core Team, R: A language and environment for statistical computing. R Foundation for Statistical Computing, Vienna, Austria. URL https://www.R-project.org/. (2021).

58. M. Simon, M. Johnson, P. T. Madsen, Keeping momentum with a mouthful of water: Behavior and kinematics of humpback whale lunge feeding. J. Exp. Biol. 215, 3786–3798 (2012).

59. S. Isojunno, D. Sadykova, S. DeRuiter, C. Curé, F. Visser, L. Thomas, P. J. O. Miller, C. M. Harris, Individual, ecological, and anthropogenic influences on activity budgets of long-finned pilot whales. Ecosphere 8, e02044 (2017).

60. S. L. DeRuiter, R. Langrock, T. Skirbutas, J. A. Goldbogen, J. Calambokidis, A. S. Friedlaender, B. L. Southall, A multivariate mixed hidden Markov model for blue whale behaviour and responses to sound exposure. Ann. Appl. Stat. 11, 362–392 (2017).

61. W. Zucchini, I. L. MacDonald, R. Langrock, Hidden Markov models for time series: An introduction using R (Chapman & Hall/CRC Press, Boca Raton, Florida, USA, 2017).

62. P. Berens, CircStat: A Matlab toolbox for circular statistics. J. Stat. Softw. 31, 1–21 (2009).

63. B. McClintock, Worth the effort? A practical examination of random effects in hidden Markov models for animal telemetry data (2020), doi:10.1101/2020.07.10.196410.

64. M. A. Hernán, J. M. Robins, Causal Inference: What If (Boca Raton: Chapman & Hall/CRC, 2020).

65. J. Pinheiro, D. Bates, S. DebRoy, D. Sarkar, R. C. Team, nlme: Linear and Nonlinear Mixed Effects Models. R Packag. version 3.1–153 (2021) (available at https://cran.r-project.org/package=nlme).

66. D. Bates, M. Mächler, B. M. Bolker, S. C. Walker, Fitting linear mixed-effects models using lme4. J. Stat. Softw. 67, 1–48 (2015).

67. J. W. Lawson, A. M. Magalhães, E. H. Miller, Important prey species of marine vertebrate predators in the northwest Atlantic: Proximate composition and energy density. Mar. Ecol. Prog. Ser. 164, 13–20 (1998).

68. D. Lüdecke, ggeffects: Tidy data frames of marginal effects from regression models. J. Open Source Softw. 3, 772 (2018).

69. J. Textor, B. van der Zander, M. K. Gilthorpe, M. Liskiewicz, G. T. H. Ellison, Robust causal inference using directed acyclic graphs: The R package “dagitty”. Int. J. Epidemiol. 45, 1887–1894 (2017).
